# Widespread mortality of trembling aspen (*Populus tremuloides*) throughout interior Alaskan boreal forests resulting from a novel canker disease

**DOI:** 10.1371/journal.pone.0250078

**Published:** 2021-04-08

**Authors:** Roger W. Ruess, Loretta M. Winton, Gerard C. Adams

**Affiliations:** 1 Institute of Arctic Biology, University of Alaska, Fairbanks, Alaska, United States of America; 2 United States Department of Agriculture Forest Service, Forest Health Protection, Fairbanks, Alaska, United States of America; 3 Department of Plant Pathology, University of Nebraska, Lincoln, Nebraska, United States of America; USDA Forest Service, UNITED STATES

## Abstract

Over the past several decades, growth declines and mortality of trembling aspen throughout western Canada and the United States have been linked to drought, often interacting with outbreaks of insects and fungal pathogens, resulting in a “sudden aspen decline” throughout much of aspen’s range. In 2015, we noticed an aggressive fungal canker causing widespread mortality of aspen throughout interior Alaska and initiated a study to quantify potential drivers for the incidence, virulence, and distribution of the disease. Stand-level infection rates among 88 study sites distributed across 6 Alaska ecoregions ranged from <1 to 69%, with the proportion of trees with canker that were dead averaging 70% across all sites. The disease is most prevalent north of the Alaska Range within the Tanana Kuskokwim ecoregion. Modeling canker probability as a function of ecoregion, stand structure, landscape position, and climate revealed that smaller-diameter trees in older stands with greater aspen basal area have the highest canker incidence and mortality, while younger trees in younger stands appear virtually immune to the disease. Sites with higher summer vapor pressure deficits had significantly higher levels of canker infection and mortality. We believe the combined effects of this novel fungal canker pathogen, drought, and the persistent aspen leaf miner outbreak are triggering feedbacks between carbon starvation and hydraulic failure that are ultimately driving widespread mortality. Warmer early-season temperatures and prolonged late summer drought are leading to larger and more severe wildfires throughout interior Alaska that are favoring a shift from black spruce to forests dominated by Alaska paper birch and aspen. Widespread aspen mortality fostered by this rapidly spreading pathogen has significant implications for successional dynamics, ecosystem function, and feedbacks to disturbance regimes, particularly on sites too dry for Alaska paper birch.

## Introduction

Climate-driven changes in the spread and virulence of plant pathogens over the past 50 years have dramatically altered the composition, successional dynamics, and function of forest ecosystems globally [1–5]. However, the complexity of host-pathogen-environment interactions across multiple scales makes understanding and predicting pathogen outbreaks difficult. At the molecular level, the encoding of plant immune receptors by disease resistance genes involves the synthesis and coordination of cell protein domains that have only partially been characterized [6–9]. At the ecological scale, pathogen outbreaks often interact with other biotic agents such as insects and vertebrate herbivores that stress tree carbon (C) balance and facilitate pathogen infections through transport or wounding [10–13]. The co-occurrence of these and other factors such as competition, stand age and drought collectively compromise plant defensive systems and significantly increase vulnerability to diseases [14–17]. Recent advances in coupling remote detection of tree mortality with landscape and climate modeling are providing insights into the mechanisms of disease spread and, in many cases, have identified drought as a contributing driver of pathogen outbreaks over the past several decades [18–21].

Widespread growth declines and mortality of trembling aspen (*Populus tremuloides* Michx.) throughout western Canada and the U.S. have been linked to drought since the late 1990s [22–25]. Drought-induced aspen dieback has interacted with outbreaks of herbivorous and wood-boring insects and fungal pathogens, resulting in a “sudden aspen decline” throughout much of aspen’s range, exacerbating sensitivity to both drought and pest and pathogen outbreaks [14,15,24,26]. Aspen is equipped with a robust system of constitutive and induced secondary chemical defenses against insects and pathogens [27]. However, multiple stressors over prolonged periods trigger a spiraling set of feedbacks between declining plant C and water balance that eventually kills trees [28,29]. There are several hydraulic adaptations that allow aspen to thrive in drier habitats; however, as we discuss below, many of these same characteristics are affected directly and indirectly by pathogens and herbivorous insects to increase vulnerability to hydraulic failure brought on by drought and freeze-thaw cycles [30–36]. This makes aspen and other poplars growing in semi-arid environments particularly sensitive to the feedbacks between drought and insect and/or pathogen outbreaks [15].

Interior Alaska has warmed more than twice as fast as the contiguous U.S. over the past 60 years [37,38]. Across interior Alaska, aspen ring-width increment has shown a gradual decline over this time period in response to temperature-induced drought stress, and more recently, declines in growth and plant water balance have been correlated with damage from an outbreak of the aspen leaf miner (*Phyllocnistis populiella* Cham.) which began in the early 2000s and is now widespread throughout the state [39–44]. Monitoring of permanent plots since the mid-1990s revealed aspen growth declines and increased mortality throughout interior Alaska attributed to recent increases in July vapor pressure deficit coupled with the aspen leaf miner outbreak [45]. In 2015, we first noticed a highly aggressive fungal canker infecting the main stem of aspen, which was widespread throughout the Alaskan boreal forest, particularly north of the Alaska Range. Although likely associated with the combined stresses of drought and the leaf miner outbreak, this “aspen running canker” appeared to be instrumental in the mortality reported. The pathogen, previously new to science, has been recently named *Neodothiora populina* Crous, G.C. Adams, & Winton [46], and we have completed a Koch’s postulates study that confirms this pathogen is causing the disease outbreak we characterize here (S1 Document) [47]. The purpose of the current study was to quantify the incidence, virulence, and distribution of the pathogen as a function of stand structure, landscape position and summer climate throughout the range of aspen within the Alaskan boreal forest. Given recent studies highlighting the interaction between C stress imposed by the nearly ubiquitous aspen leaf miner and climate warming [39,42,43,45], we were particularly interested in how stand, landscape, and climatic factors interacted to influence the variability in disease incidence and related mortality across the regional scale.

## Materials and methods

### Study sites

**Fig 1:**
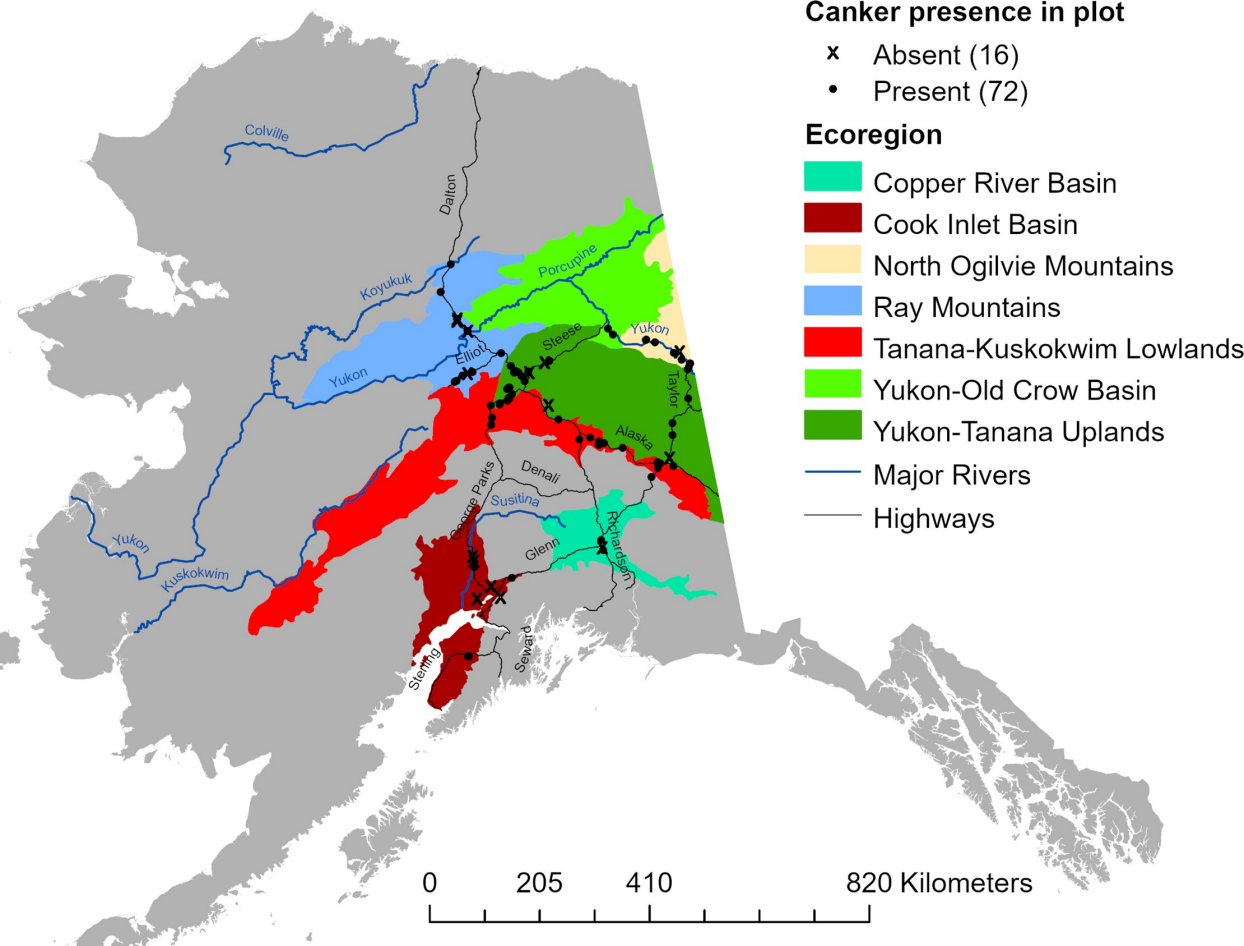
Map of study sites. Location of 88 sites inventoried for aspen canker. For analyses, sites within the Yukon-Old Crow Basin were grouped with those from the North Ogilvie Mountains.

The incidence of running stem canker in aspen was inventoried within previously established networks of long-term monitoring and inventory plots scattered across interior Alaska, and within additional sites selected specifically for this study (Fig 1, DOI: 10.6073/pasta/85f4357d25255ed1373e4d2afeac4784). These included sites within the Bonanza Creek (BNZ) Experimental Forest (BCEF) and sites within the BNZ Regional Site Network (RSN), both maintained by the BNZ Long-Term Ecological Research program (BNZ LTER). Forest inventory plots within the BCEF were established in 1987 to study boreal forest vegetation and ecosystem change following fire in uplands and flooding along the Tanana River near Fairbanks, Alaska. Stands range in age from 40 to 200 years old, and are dominated by a mix of hardwoods including aspen, Alaska paper birch (*Betula neoalaskana* Sarg.), and balsam poplar (*Populus balsamifera* L.), and conifers including white spruce (*Picea glauca* (Moench) Voss), black spruce (*Picea mariana* (Mill.) B.S.P.), and tamarack (*Larix laricina* (Du Roi) K. Koch). Sites within the RSN were established in 2013 to study long-term vegetation and ecosystem responses across a large regional scale to the recently intensified fire regime driven by climate warming throughout interior Alaska. When established, RSN sites were characterized as young (< 15 yrs), intermediate age (40–60 yrs), or mature (> 80 yrs), dominated by the same tree species found within BCEF with stand composition being a function of site conditions and successional trajectory. Study plots within the BCEF and RSN networks are 50 x 60 m, and long-term inventory data on vegetation, climate and soils can be found on the BNZ LTER webpage (http://www.lter.uaf.edu/data/data-catalog). We also surveyed plots within the Cooperative Alaska Forest Inventory (CAFI) network, a network of 203 sites representing a wide range of mature boreal forest stands scattered across interior and south-central Alaska including the Kenai Peninsula [48]. Each CAFI site includes three 0.1-acre plots which we combined into one plot for each site. Only sites containing aspen within LTER and CAFI networks were inventoried. We established additional plots within stand types throughout interior Alaska that were underrepresented by the CAFI and LTER plots in order to encompass a broader range of aspen stand ages and overstory compositions. These “NEW” plots (~0.08 ha) included intermediate-age to mature stands within the Yukon Old Crow Basin and North Ogilvie Mountains ecoregions along a 254 km stretch of the Yukon River between Circle and Eagle River, Alaska, and younger stands established within areas along the road system (always > 200 m from the road) that burned between 1980–2000. In total, these 88 study sites encompassed a minimum boundary area of over 209,000 km^2^.

#### Canker inventory

All standing aspen trees within each plot were recorded as live or dead, diameter measured at 1.37 m above the ground (DBH) and evaluated from bottom to top for damages. On CAFI and LTER plots, up to three damage agents from a standardized list were recorded in order of severity (i.e., agents threatening survival being more important than agents that reduce growth or wood quality). These included weather-related breakage or blowdown, insect (including aspen leaf miner) or other pathogen damage such as decay and fungal conks, moose browsing or bark stripping, etc. Running canker lesions were identified by discolored, often sunken bark with distinct margins, and necrosis of cambium with unusual length (>10 cm) often girdling the stem but exhibiting no fungal fruiting bodies or the smell of bacterial fermentation. The aspen stands examined were remarkably free of any cankers exhibiting the typical diagnostic characteristics of well-known aspen pathogens encountered at high frequency in the Lake States and southern Rocky Mountain, such as distinctive fruiting bodies, black stroma, pustules, cirrhi, fruity smell, colored oozing, or target-shaped rings of callousing.

Where possible, the likely cause of death was recorded for standing dead trees. A severity rating for each damage agent was assigned based upon the percentage of affected plant tissue as follows: 0, none observed; 1, trace to 5% affected area (i.e., roots, bole circumference, foliage, branches, buds); 2, 6–15%; 3, 16–35%; 4, 36–67%; 5, 68–100%. The location on the tree was also recorded for each damage agent. On “NEW” plots, only live/dead, DBH, canker status, leaf miner status, and moose browse damages were recorded.

### Statistics

The probability of canker infection (*π*) for an individual tree was modeled using mixed effects linear logistic regression using a binary response model of the form:

$$logit\;(\pi )=\log\;(\frac{\pi }{1-\pi })=\alpha +\beta^{\prime }x^{\prime }+\gamma$$

where *β’* = (*β*_*1*_, *…*., *β*_*s*_*)* is the vector of s slope parameters for *x*’ (= *x*_*1*_, *…*, *x*_*s*_) predictor variables (fixed effects), and γ represents a single random effect. We first fit a global model that included tree DBH (cm), other damage agents, a suite of stand-level structural and landscape variables, and ecoregion as fixed effects, to assess whether site (numbered 1–88) or site within ecoregion was the best single random intercept effect. This allowed for an assessment of the variation in canker infection among ecoregions while accounting for the potential autocorrelation among sites. Stand-level variables included aspen density (trees ha^-1^), aspen basal area (m^2^ ha^-1^), average aspen DBH for the stand (cm), and relative aspen density (% of all trees that were aspen). Leaf damage by the aspen leaf miner was not included as a fixed effect because all sites had high and indistinguishable levels of herbivory by leaf miners (severity class 5). Landscape variables for each site included slope, elevation, and a derived variable ranging from 0 to 180° representing the degree of southerly exposure. We tested for multicollinearity among independent variables by calculating variable inflation factor (VIF) values for each predictor using separate linear regressions among predictor variables, using a cutoff for acceptance of VIF < 2.0 for inclusion in models. Models were fitted using the *glmer* function (*lme4* package) in R statistical software version 3.4.2 [49]. Comparisons of models with different fixed effects were made using maximum likelihood, with AIC values compared using an analysis of deviance test using the *anova* function (*lmerTest* package) before refitting models using restricted maximum likelihood. For models with AIC values differing by < 2, the one with fewer parameters was selected. Pairwise comparisons between ecoregions were made using the *glht* function (*multcomp* package). Because the number of sites varied among ecoregions and sites were not randomly distributed within ecoregions, we caution that these analyses may not necessarily allow true inference at the scale of the ecoregion. We report marginal (*R*^*2*^_*m*_) and conditional (*R*^*2*^_*c*_) values for models to evaluate variance explained by fixed and fixed + random effects, respectively (*r*.*squaredGLMM* function in *MuMin* package). Model parameters are reported in equation form, and graphically presented as odds ratios for fixed effect parameters, estimated by exponentiating the corresponding parameter estimate, along with 95% Wald confidence intervals.

To determine whether variation in summer climate contributed to spatial differences in canker infection, we optimized logistic models using climate variables and stand-level parameters as fixed effects and one of several random effects to account for non-independence at potentially multiple spatial scales. Ecoregion and climate variables were not considered as fixed effects in the same model because discriminant function analysis (PROC DISCRIM, SAS) [50] revealed that sites could be classified into ecoregions with a high degree of accuracy using climate variables, suggesting these variables were potentially confounded (see below). Model selection involved first optimizing the random effect structure by comparing global models that included all possible fixed effects plus either site, ecoregion, or site within ecoregion as the random effect, followed by model optimization of fixed effects as described above. Landscape and stand-structural characteristics were derived from our stand-level inventory data sets, and comparison of *R*^*2*^_*m*_ and *R*^*2*^_*c*_ values enabled assessment of variance explained by fixed and fixed + random effects. Average mean monthly precipitation (mm), air temperature (°C), vapor pressure (Pa), and frost-free growing season length (days) were obtained from the Scenarios Network for Alaska and Arctic Planning (SNAP) as downscaled and spatially interpolated climate data for 1980–2016 (https://www.snap.uaf.edu/tools/data-downloads). These climate data are estimates of historical monthly climatic variables for any given locale in Alaska downscaled to a 1-km grid resolution using PRISM (Parameter–elevation Relationships on Independent Slopes Model) which integrates location, elevation, coastal proximity, topographic variables, vertical atmospheric layer, and orographic effectiveness of the terrain [51]. We calculated vapor pressure deficit (VPD; hPa) as the difference between monthly saturation vapor pressure and monthly vapor pressure after calculating saturation vapor pressure from mean monthly temperature [52]. We were particularly interested in VPD because of the reported drought sensitivity of aspen to VPD throughout interior Alaska [45] and the known sensitivity of other cankers to variation in water stress [53]. Analyses revealed a high degree of collinearity in VPD among summer months; therefore, logistic models incorporating climate variables were run for each month separately, where the inclusion of VPD, precipitation and growing season length resulted in VIF < 2. All data reported throughout the manuscript are means ± 1 SE.

## Results

### Stand structure

**Fig 2:**
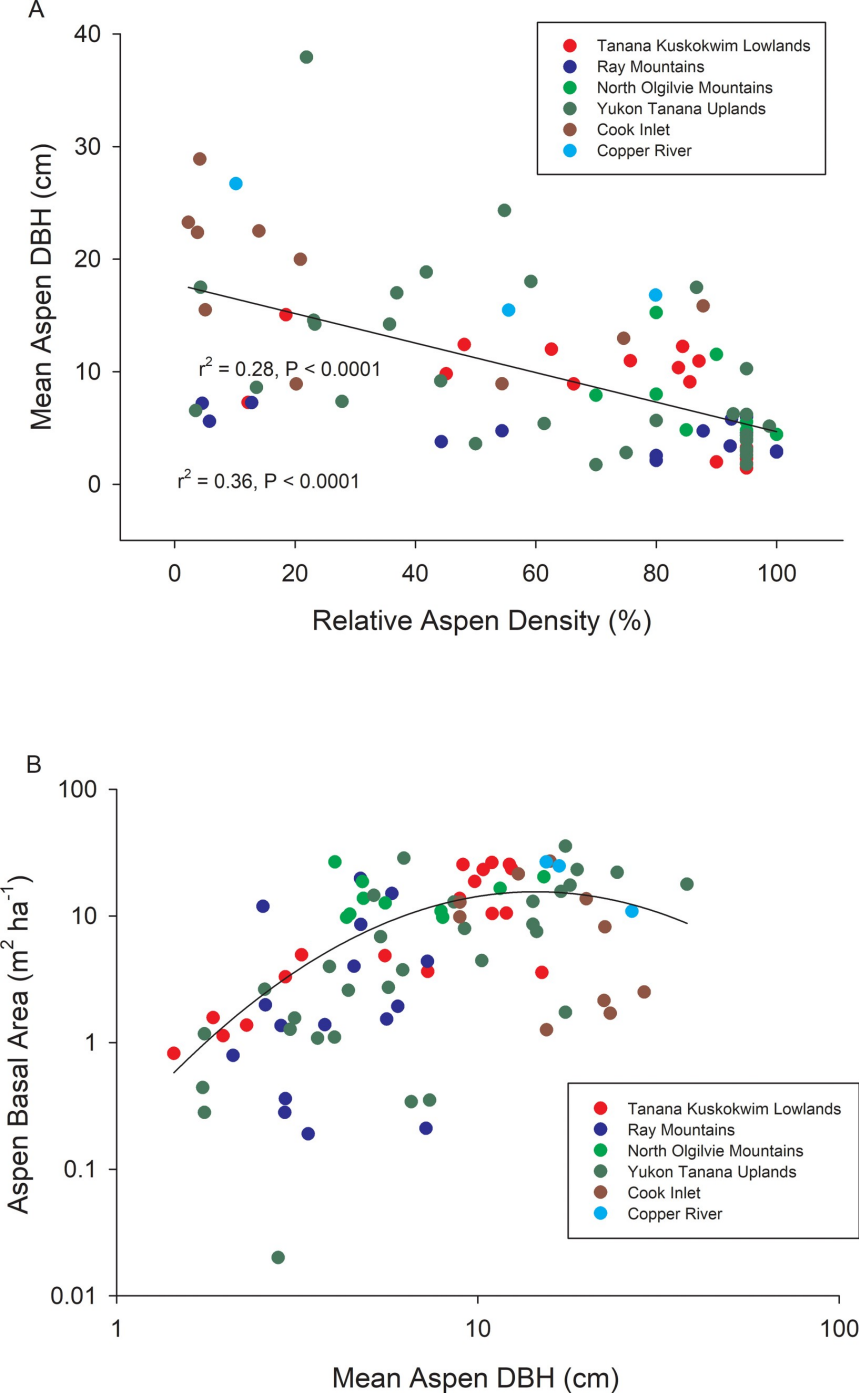
Relationships between stand structural characteristics across the 88 study sites. Mean aspen DBH vs. percentage of trees that were aspen (= relative aspen density) across stands where aspen grew with white spruce, black spruce, Alaskan paper birch, or a combination (A) and aspen basal area vs. mean aspen DBH (B) across study sites.

A total of 16,576 aspen trees were surveyed across 88 study sites located within 6 ecoregions (Fig 1, Table 1). Relative aspen density and mean aspen DBH were inversely correlated across the 88 study sites, reflecting the dominance of smaller-diameter trees in early successional stands (Fig 2A). Stand-level aspen basal area increased non-linearly with mean aspen DBH, indicating that although big trees have an influence on stand basal area, stands with a higher density of intermediate-sized trees frequently show the highest levels of aboveground biomass (Fig 2B). Stand age was not assessed, but it is likely that mean aspen DBH increased in older stands, albeit at different rates given the range of landscape positions and stand compositions that were sampled. Aspen density (P < 0.05), relative aspen density (P < 0.001), stand-level aspen basal area (P < 0.01), and mean aspen DBH (P< 0.0001) all varied among ecoregions, primarily due to a low density of large aspen trees at Copper River and Cook Inlet sites (Table 1). Despite these apparent ecoregion differences in stand structure, we found a relatively high average error rate (55.3%) when discriminant function analysis (PROC DISCRIM, SAS) was used to classify stands by ecoregion based only on these 4 stand structural characteristics. Classification error rates were particularly high for the Tanana Kuskokwim Lowlands (83.3%), Yukon Old Crow Basin (60.0%), and Yukon Tanana Uplands (90.3%). In contrast, a similar number of climate variables (see below) produced a relatively low average error rate (6.9%) when classifying sites by ecoregion, indicating that climatic differences were a stronger diagnostic than stand composition when grouping sites by ecoregion. This is to be expected because our sites were not randomly distributed within ecoregions, although they were geographically distinct. Summaries for salient climate variables are shown in S1 Fig.

**Table 1:** Aspen structural characteristics of 88 stands averaged by ecoregion.

Ecoregion	Number of Sites	Aspen Density (trees ha^-1^)	Relative Aspen Density (%)	Aspen BA (m^2^ ha^-1^)	Mean Aspen DBH (cm)
Cook Inlet	10	865 ± 317 b	29 ± 10 c	10.0 ± 2.8 abc	17.9 ± 2.1 a
Copper River	3	945 ± 361 b	49 ± 20 bc	20.8 ± 5.0 a	19.7 ± 3.5 a
Ray Mountains	16	2232 ± 638 b	71 ± 9 ab	4.6 ± 1.5 c	4.3 ± 0.4 c
Tanana Kuskokwim Lowlands	18	2434 ± 319 b	74 ± 6 ab	11.2 ± 2.3 ab	7.7 ± 1.0 bc
Yukon Tanana Uplands	31	1582 ± 348 b	63 ± 6 b	8.4 ± 1.7 bc	9.6 ± 1.7 b
North Ogilvie Mountains	10	4273 ± 1570 a	89 ± 3 a	14.9 ± 1.8 a	7.1 ± 1.2 bc

Two sites from the Yukon-Old Crow Basin ecoregion were combined within the North Ogilvie Mountains ecoregion for statistical purposes as all 10 sites along the stretch of the Yukon River between Circle and Eagle River were within similar landscapes. Values within columns with different letters indicate significant differences among ecoregions for variables at P < 0.05 (ANOVA).

### Canker incidence

**Fig 3:**
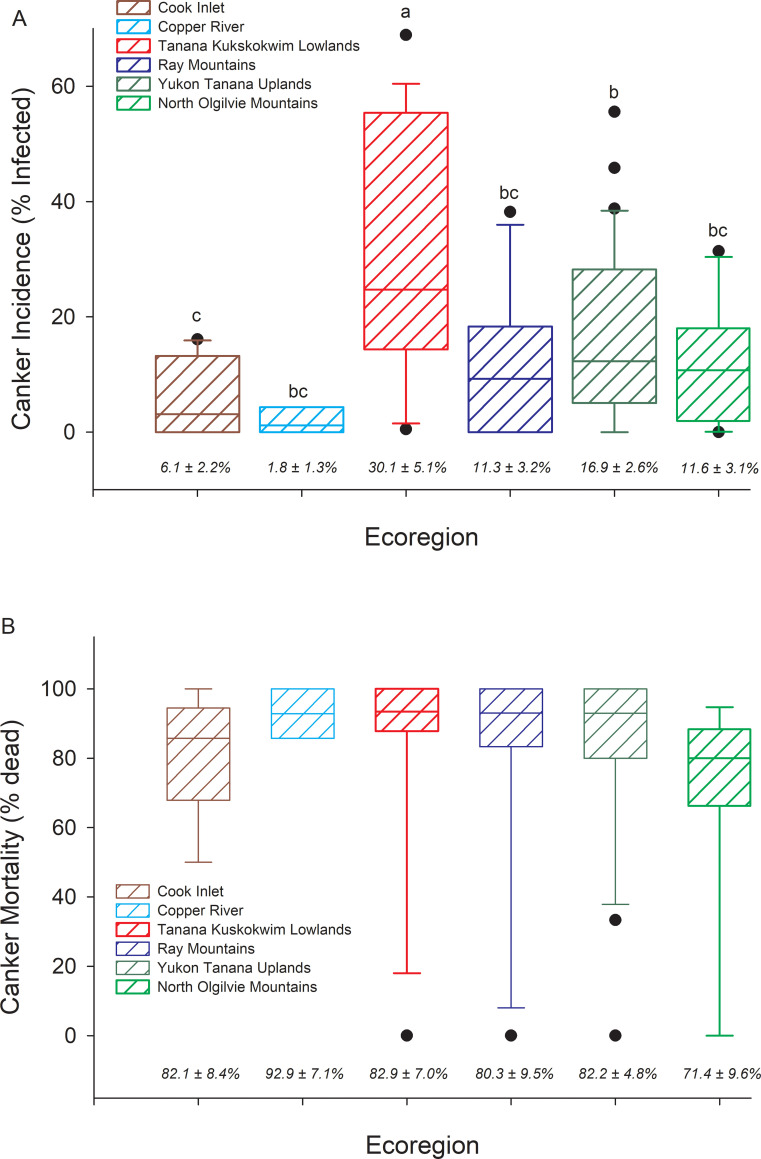
Canker incidence and canker mortality across ecoregions. Box plots of (A) canker incidence (% aspen trees infected) by ecoregion, and (B) % of trees infected with canker that are dead. Ecoregions sharing different letters are significantly different at P < 0.05 (ANOVA model, F_5,82_ = 5.10, P < 0.001). Means ± 1 STD error are listed across the bottom of the graph.

**Fig 4:**
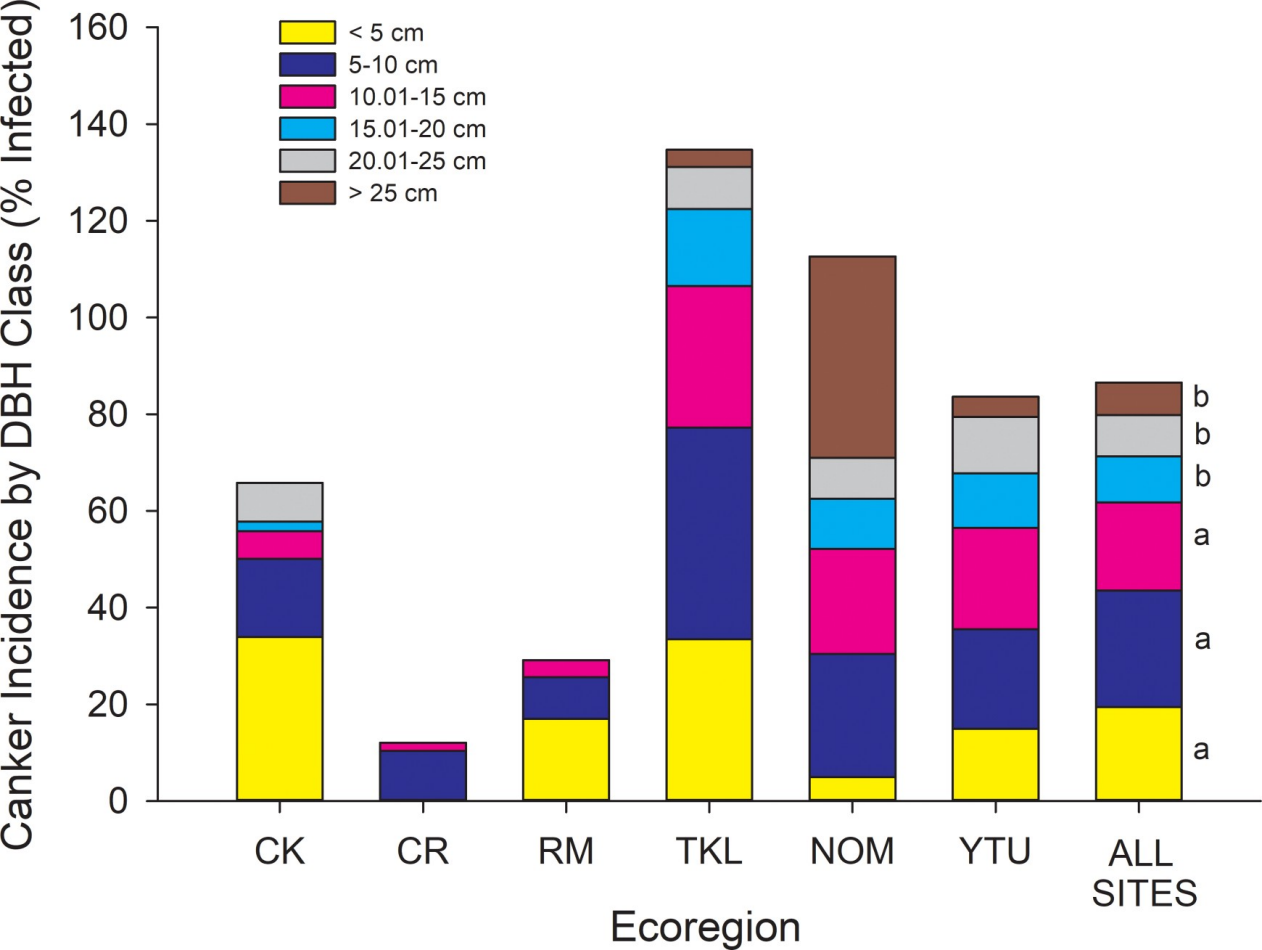
Incidence of canker infection by DBH class for each ecoregion and as an average across all 88 sites (last column). For the last column, size classes sharing similar letters are not different at P < 0.05 (ANOVA, F_5,291_ = 5.42, P < 0.0001). CK = Cook Inlet, CR = Copper River, RM = Ray Mountains, TKL = Tanana Kuskokwim Lowlands, NOM = North Ogilvie Mountains, YTU = Yukon Tanana Uplands. The absence of size classes within ecoregions is because no trees were found within that size class, or that none of the trees within that size class were infected.

**Fig 5:**
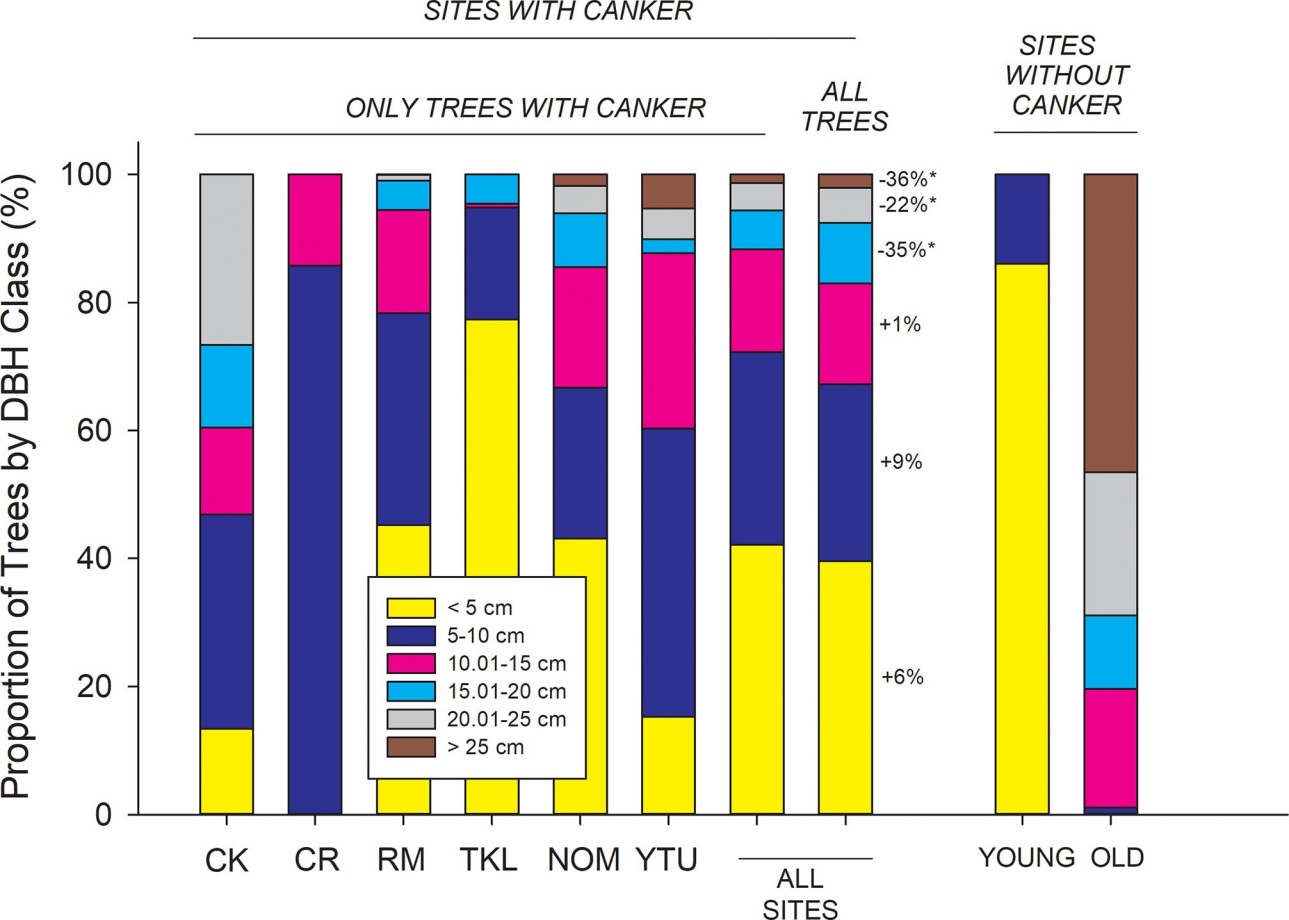
Proportion of DBH size classes at 72 sites with canker (columns 1–8) and at 16 sites without canker (columns 9–10). For sites with canker, the first 6 columns show data for only trees with canker categorized by ecoregion, and column 7 shows the mean across all sites for trees with canker. Column 8 shows the size distribution of all trees (with and without canker) at sites where canker was present. Values beside size classes in Column 8 refer to the % increase in proportion of infected trees vs. the population as a whole for each size class (*P < 0.10). For example, there are 6% more trees infected with canker in DBH class 1 than there are in the population (NS), while there are 35% fewer trees infected with canker in 15–20 cm size class than there are in the population as a whole (P < 0.10). Columns 9 and 10 show data for sites without canker, broken down into young sites (n = 9) and older sites (n = 7). For sites without canker, young sites (column 9) were located within recent burn perimeters scattered across the Yukon Tanana Uplands, Ray Mountains, and North Ogilvie Mountains ecoregions, while 6 of 7 old sites (column 10) were located south of the Alaska Range within the Cook Inlet and Copper River ecoregions. CK = Cook Inlet, CR = Copper River, RM = Ray Mountains, TKL = Tanana Kuskokwim Lowlands, NOM = North Ogilvie Mountains, YTU = Yukon Tanana Uplands.

Canker was present at 72 of the 88 sites inventoried. The proportion of trees infected with canker ranged from 1.8 ± 1.3% at Copper River sites to 30.1 ± 5.1% at Tanana Kuskokwim Lowlands sites, where infection was highest on average (P < 0.001) and ranged from 0.5% to 68.9% across sites (Fig 3A). The proportion of trees with canker that were dead was high and invariant across ecoregions, averaging above 70% (Fig 3B). However, even within interior Alaskan ecoregions where infection was high, some sites showed no mortality of infected trees, suggesting the disease was at early developmental stages or perhaps trees had high resistance against the canker in some stands. Across all 88 sites, the incidence of canker infection was significantly greater in smaller diameter trees than larger diameter trees (Fig 4). The exception to this was within the North Ogilvie Mountains, where 3 of 4 trees > 25 cm DBH at one site were infected, increasing the overall cross-site infection rate in the largest trees. For the 72 sites where canker was detected, higher canker incidence in smaller DBH trees and lower incidence in larger DBH trees occurred to greater extents relative to all trees within the population (Fig 5, comparing columns 7 & 8). Canker was not found at 16 sites, which were located either within recent burn perimeters (< 15 years old) north of the Alaska Range, or in older stands with a high proportion of large trees south of the Alaska Range. Thus, while younger trees in intermediate-aged stands had high infection rates, younger trees in younger stands had little to no canker infection.

### Modeling canker incidence

Model selection revealed that the best mixed-effect model estimating the probability of canker infection across ecoregions included tree DBH, stand structural parameters (aspen basal area, relative aspen density, and aspen density) and ecoregion as fixed effects, and site as a random effect. Values for *R*^*2*^_*m*_ (0.23) and *R*^*2*^_*c*_ (0.45) indicated that fixed effects and the random effect of site, accounting for the autocorrelation among sites, contributed similarly to total explained variance:

$$\begin{array}{l} \mathrm{Logit}\;(\pi )=\log\;(\pi /(1‐\pi ))=-0.761-0.177*DBH-0.023*\mathrm{Relative}\;\mathrm{Aspen}\;\mathrm{Density}+0.092*\mathrm{Aspen}\\ \mathrm{Basal}\;\mathrm{Area}+0.106*\mathrm{Mean}\;\mathrm{Aspen}\;DBH-1.413*CK-3.263*CR-0.358*RM+0.692*TKL-\\ 0.736*\mathrm{NOM} \end{array}$$

**Fig 6:**
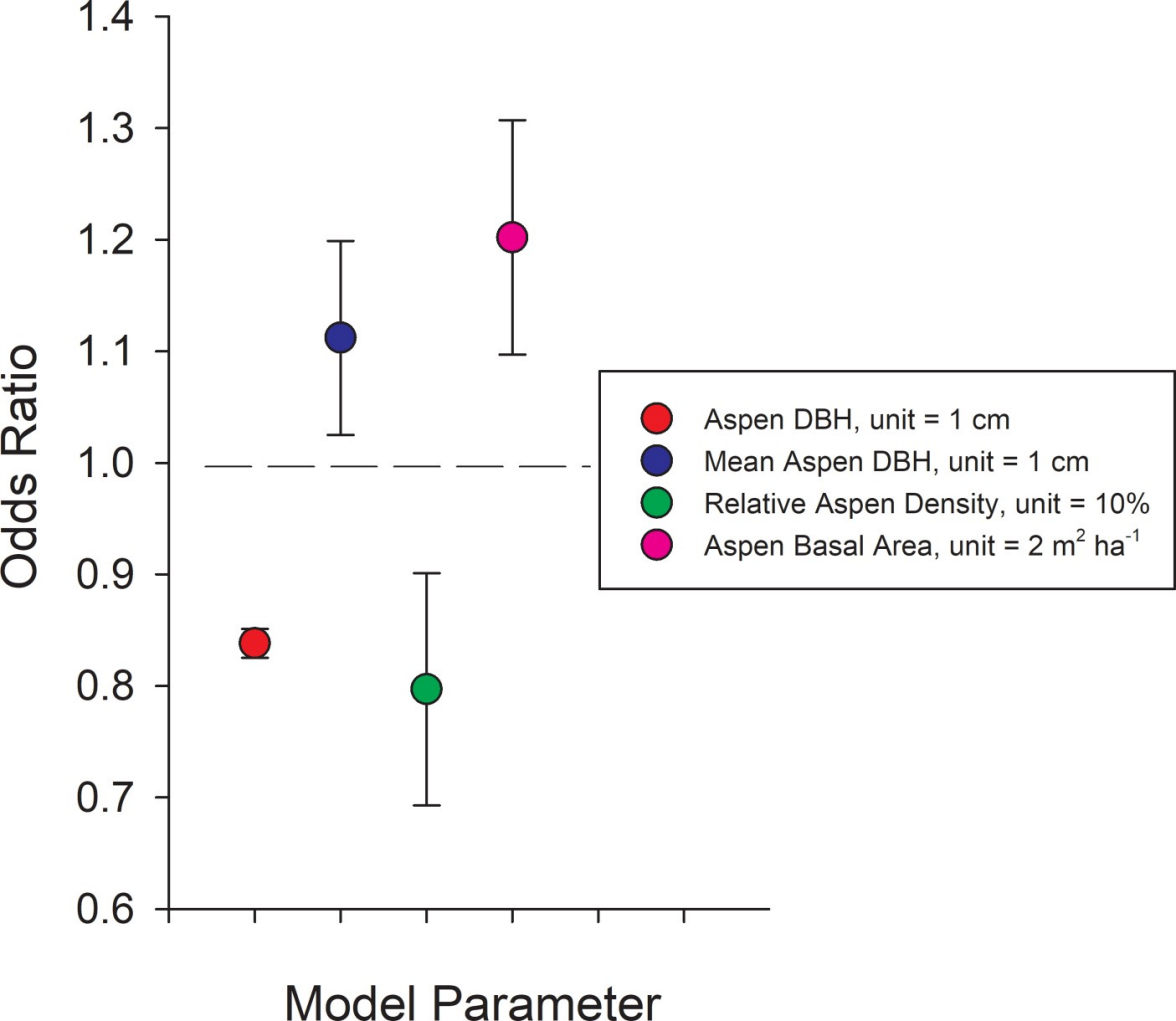
Modeling canker incidence based on stand parameters. Odds ratios for stand structural parameters from the mixed-effects logistic model: Logit (π) = log (π/(1- π)) = –0.761–0.177*DBH– 0.023*Relative Aspen Density + 0.092*Aspen Basal Area + 0.106*Mean Aspen DBH– 1.413*CK– 3.263*CR—0.358*RM + 0.692*TKL– 0.736*NOM. Ecoregion β values are computed relative to *YTU* which does not appear in the model and has a log (odds) = the intercept when all other factors are 0. Variables with odds ratios significantly greater than 1.0 indicate a significant increase the odds of canker infection while those below 1.0 indicate a significant reduction in the probability of canker infection. For example, an odds ratio of 0.838 (red dot) means that for every 1 cm increase in DBH of a given aspen tree, the odds of having canker is reduced by 16.2% (= (1–0.839)*100), while an odds ratio of 1.112 (blue dot) means that every 1 cm increase in average stand-level DBH (a surrogate for stand age) increased the odds of having canker by 11.2% ((1.112–1)*100). Ecoregion abbreviations follow Fig 4.

**Fig 7:**
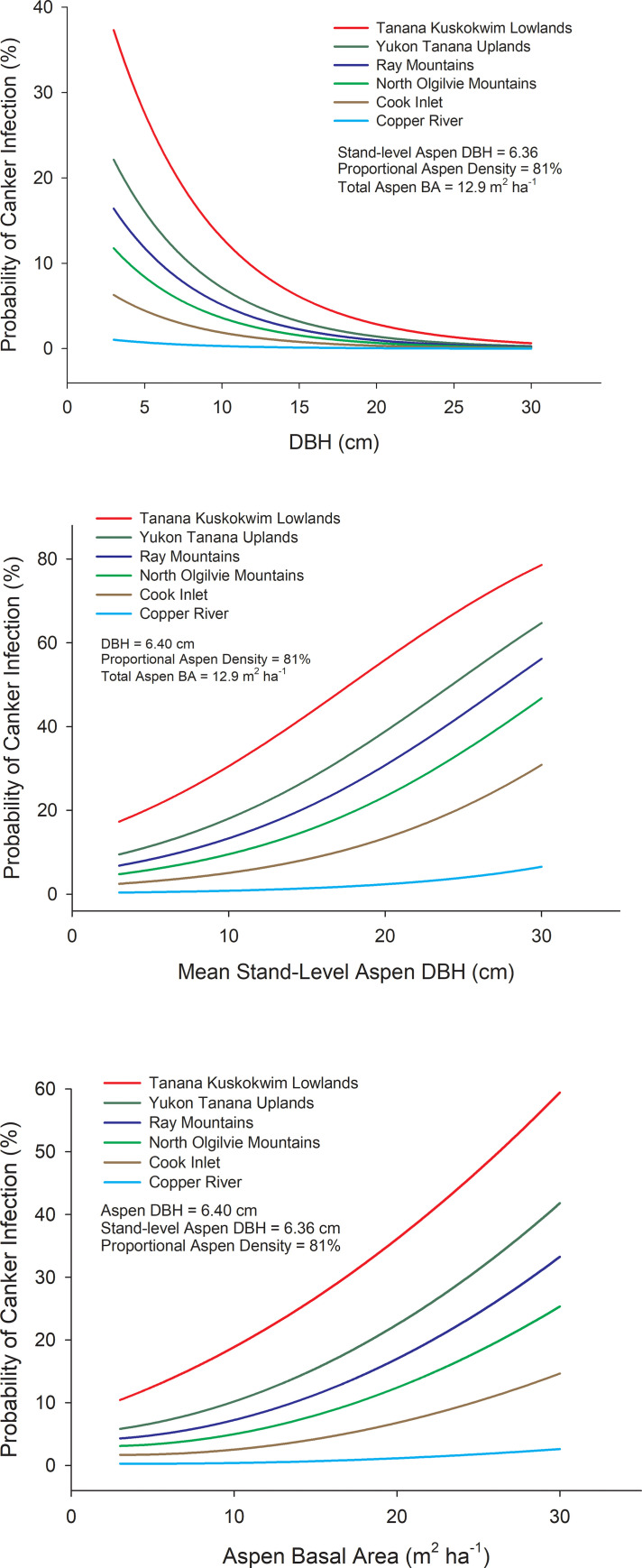
Modeling canker incidence among ecoregions. Probability of canker infection for different ecoregions as a function of DBH of an aspen tree (A), mean stand-level aspen DBH, a surrogate for stand age (B), and aspen basal area (C). Probabilities were calculated by fixing all other variables as mean values using the following equation derived from a mixed-effects logistic regression model incorporating site as a random effect: Logit (π) = log (π/(1- π)) = –0.761–0.177*DBH– 0.023*Relative Aspen Density + 0.092*Aspen Basal Area + 0.106*Mean Aspen DBH– 1.413*CK– 3.263*CR– 0.358*RM + 0.692*TKL– 0.736*NOM. Ecoregion β values are computed relative to *YTU* which does not appear in the model and has a log(odds) = the intercept when all other factors are 0. Contrasts derived from logistic models indicated the following differences in canker incidence among ecoregions; *TKL*a, *YTU*ab, *RM*bc, *NOM*abc, *CK*cd, *CR*d, where ecoregions with different letters are significantly different at P < 0.05. Ecoregion abbreviations follow Fig 4.

Ecoregion abbreviations (see Fig 4) were coded as 1 or 0 in the model. Ecoregion β values are computed relative to the Yukon Tanana Uplands (*YTU*) which does not appear in the model and has a log (odds) = the intercept plus other stand-level factors when all binary variables for ecoregions = 0. One way to visualize the effects of the selected stand variables is by examining how a unit change of a given variable affects the odds ratio of canker infection when all other variables are held constant (Fig 6). For example, a 1 cm increase in the DBH of an aspen tree decreased the odds of canker infection by 16.2%, while a 1 cm increase in average aspen DBH (a surrogate for stand age) increased the odds of canker infection by 11.2%. Thus, the model shows that probability of canker infection is highest in smaller diameter trees growing in older stands. Increasing stand level aspen basal area by 2 m^2^ ha^-1^ increased the odds of canker infection by 20.2% and increasing the relative aspen density by 10% reduced the odds of canker infection by 20.3%. This inverse relationship between canker incidence and relative aspen density is likely driven by little to no canker in young trees within recent burn perimeters. Variability in elevation, slope or southerly exposure did not significantly influence the odds of canker infection. Differences among ecoregions in the probability of canker infection were driven by high canker incidence in the Tanana Kuskokwim Lowlands and low incidence of canker south of the Alaska Range, which are similar to differences in canker incidence calculated directly from survey data (Fig 7).

### Incorporating climate variables into modeling canker incidence

**Fig 8:**
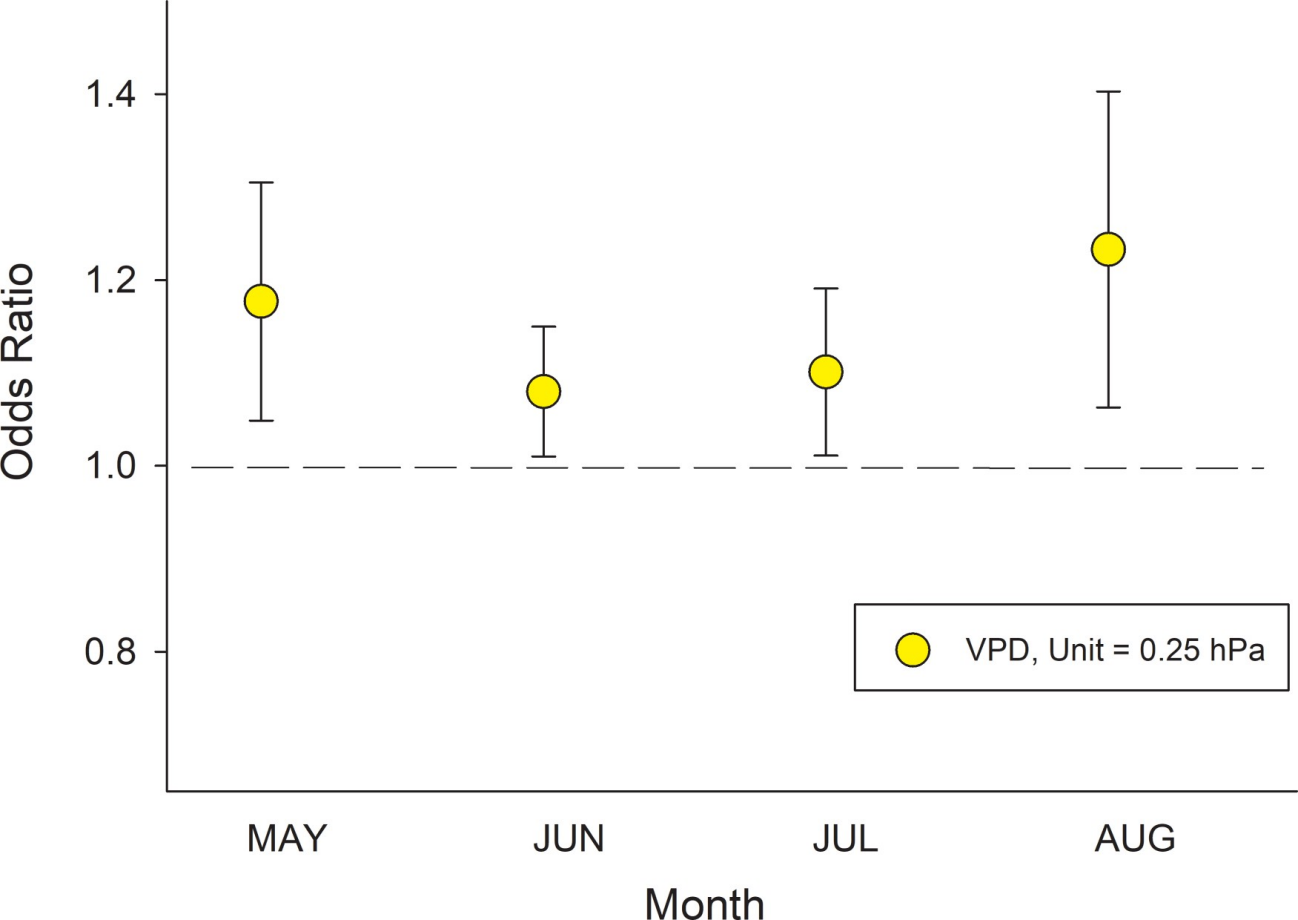
Modeling canker responses to variations in stand structure and climate. Odds ratios for canker incidence response to 0.25 hPa increases in VPD during May, June, July and August derived from the mixed-effects logistic equation model: Logit (π) = log (π/(1- π)) = α + β_1_ *DBH + β_2_ *Relative Aspen Density + β_3_ *Aspen Basal Area + β_4_ *Mean Aspen DBH + β_5_ *VPDmonth. Model results for stand structural variables and monthly VPD are show in S1 Table. Unit increases in variables with odds ratios above 1.0 significantly increase the probability of canker infection while those below 1.0 reduce the probability of canker infection. Thus, an odds ratio of 1.177 for VPD in May indicates that for every 0.25 hPa increase in VPD the odds of having canker increases by 17.7%.

The best model incorporating climate variables to predict canker infection included monthly VPD and stand structural parameters as fixed effects, and site nested within ecoregion as the random effect. Other climate variables (precipitation and growing season length) and site variables (elevation, slope and southerly) were eliminated during model optimization. The effects of stand structure variables were consistent among models incorporating VPD run separately for May, June, July, and August, and similar to those reported above where ecoregion was included as a fixed effect (S1 Table). Sites with higher monthly VPD consistently had a higher canker infection whereby increases in VPD by 0.25 hPa during May, June, July and August increased the odds of canker infection by 17.7%, 8.0%, 10.1% and 23.3%, respectively (Fig 8).

Climate sensitivity of canker infection involves a complex of factors affecting both the plant and the pathogen, as discussed below. Monthly VPD and temperature values are positively correlated during the growing season, as expected, since temperature is included in the calculation of VPD. Interestingly, VPD and precipitation are also positively correlated, albeit weakly, for May, June, and July (S2 Fig). In our global model that included all climate variables as fixed effects, increases in precipitation tended to decrease canker infection in May and August, and increase canker infection in June and July; however, precipitation was eliminated as a factor during model optimization. Although speculative, it may be that during May and August, higher rainfall improves plant water balance at some sites and thereby reduces vulnerability to canker despite the dominant net effect of warmer conditions (higher VPD) favoring pathogen growth [54,55]. But during June and July, the period of most rapid tree growth, both warmer (higher VPD) and wetter (higher PPT) site conditions appear to be benefitting pathogen growth. Overall, models show that VPD alone was the most important climate driver influencing the probability of canker infection, which is consistent with aspen drought sensitivity to the recent summer drying in interior Alaska [45]. Data from the main BNZ LTER weather station show that while average VPD is similar for May, June and July, precipitation steadily increases throughout the summer. However, soil moisture declines significantly from May through July, suggesting that trees are controlling stand water balance and potentially becoming progressively water limited despite this increase in precipitation (S3 Fig). Interesting, even when precipitation has increased substantially relative to VPD in August, sites with higher VPD continue to have higher rates of canker.

## Discussion

### Disease epidemiology and spread

The aspen mortality we and others have observed throughout interior Alaska appears to differ from the “sudden aspen decline” syndrome (*sensu* [14,15,24,56]) in the contiguous U.S. in the consistent presence of a large, fast-expanding diffuse canker, the absence of extensive branch dieback preceding mortality, and the lack of direct involvement of secondary insect pests and pathogens. We recognize the canker pathogen found on aspen in Alaska to be the primary agent of mortality (S1 Document), as such it differs from a forest decline in which biotic agents are secondary contributing factors to a primary inciting factor such as drought. Marchetti et al. (2011) [56] reported that a group of interchangeable secondary insects including borers (*Agrilus liragus* and *Saperda calcarata)*, bark beetles (*Procryphalus mucromatus* and *Trypophloeus populi*), and possibly others distinguished the Rocky Mountain decline mortality, which are not involved in the mortality driven by *N*. *populina* in interior Alaska. However, as we discuss below, we believe drought and the ubiquitous aspen leaf miner may have contributed to the *N*. *populina* outbreak by stressing the host trees. To our knowledge, the size and speed of expansion of *N*. *populina* exceeds that reported for any known fungal canker pathogen of trees in temperate or boreal forests and generally results in tree death within 1–2 years. As such, *N*. *populina* is more aggressive and severe than other aspen canker diseases, such as *Hypoxylon* canker, which is similar in size and lethality but usually takes several years to kill trees and always presents evident black stroma [53]. *Cytospora* canker pathogens (*C*. *chrysosperma*, *C*. *notastroma*, and *C*. *nivea)* were occasionally observed on fallen aspen as secondary colonizers of the *N*. *populina* cankers, as was also reported in sudden aspen decline [56]. *Cytospora* are readily differentiated by the consistent habit of producing numerous aggregated fruiting bodies on the face of annual cankers.

Across all plots where canker was found, 34% of all aspen trees were dead, of which 53% were identified as having canker; however, we suspect we underestimated the canker contribution to mortality because the disease is often difficult to identify on dead trees. All aspen trees within the CAFI and LTER site networks are individually tagged, and our inventory included all standing and down dead trees that could be located. However, trees were not recorded as having canker unless we could unequivocally identify symptoms of this particular fungal canker. We suspect that the values reported for overall disease incidence in Fig 3A are also conservative because canker symptoms may have been within the upper canopy but not visible from the ground. Moreover, given how rapidly the canker spreads within a tree, many trees likely had developing canker but undetectable symptoms.

**Fig 9:**
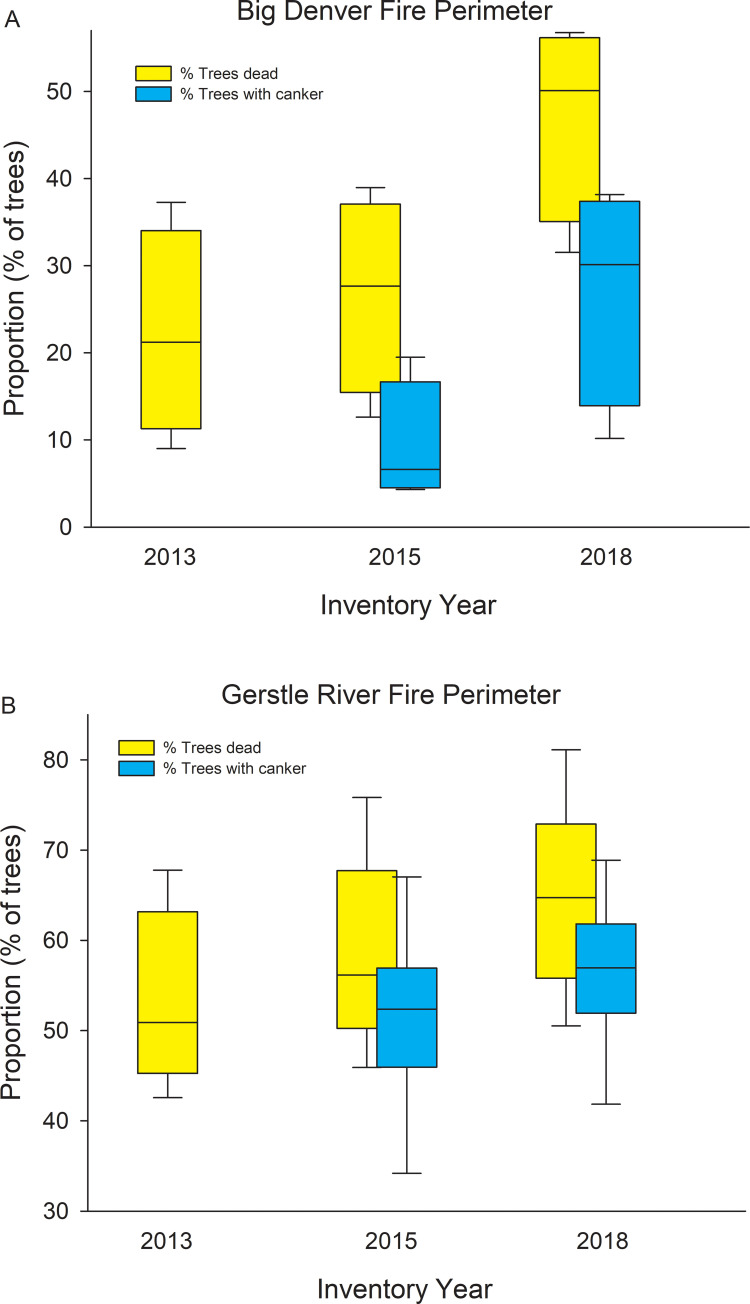
Changes in disease incidence at 3 assessments across multiple sites within 2 regions. Box plots showing % of all aspen trees dead (2013, 2015, and 2018) and % of all aspen trees with canker (2015 and 2018) for sites within the Big Denver Fire perimeter (A, n = 4 sites, Ray Mts. ecoregion) and Gerstle River Fire perimeter (B, n = 6 sites, Tanana Kuskokwim Lowlands ecoregion).

**Fig 10:**
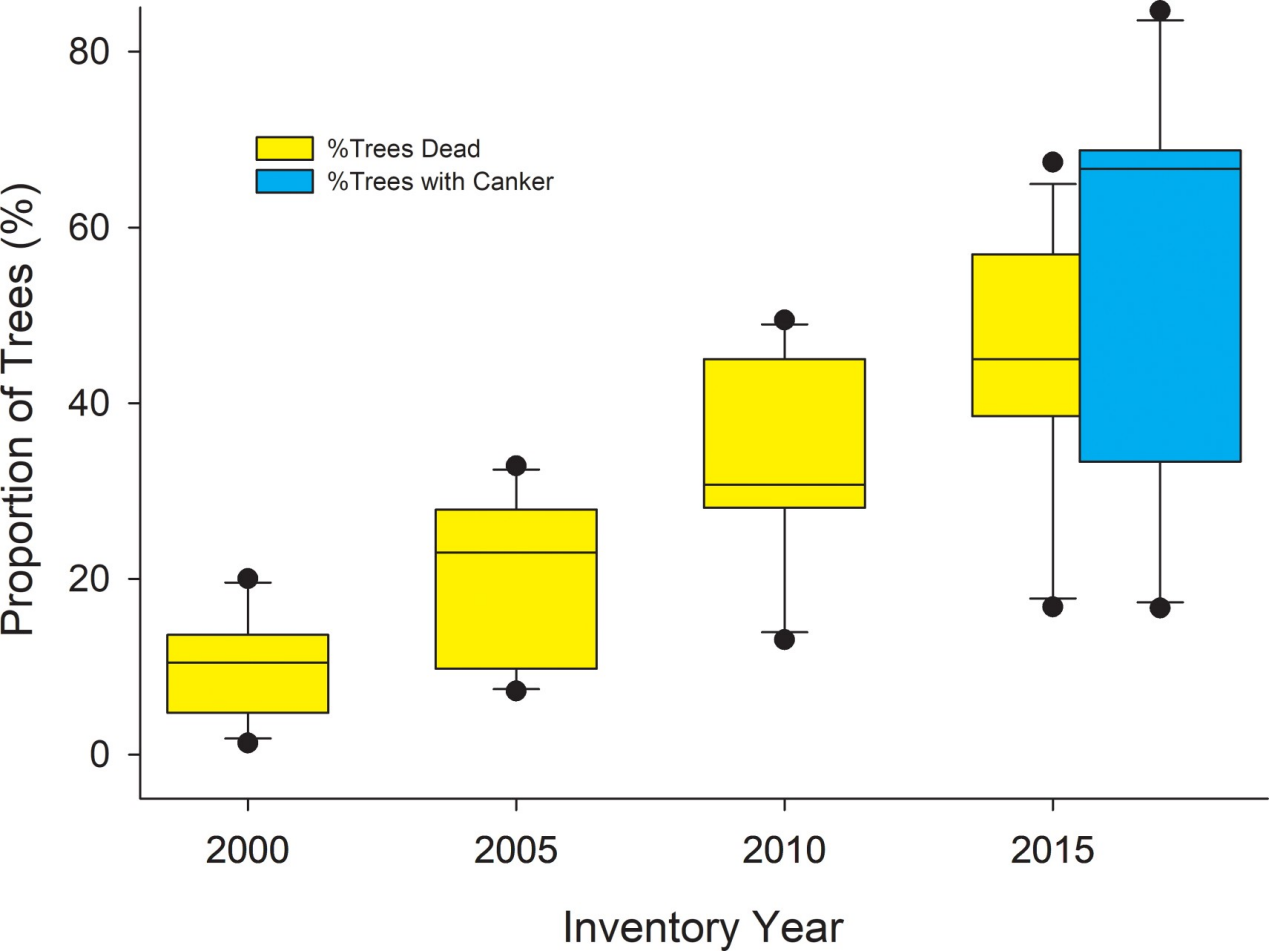
Box plots showing % of all aspen trees dead at select CAFI sites in 2000 and every 5 years thereafter. When plot inventory began in 1995, dead trees were not inventoried, but all live trees were tagged and those that subsequently died were inventoried on subsequent dates. The CAFI program included other sites in the inventory program from 1996–2013, but the 12 sites here represent the cohort of sites with the longest sampling history (other than 4 sites first inventoried in 1994 but not included here). Also shown are the proportion of trees with canker at these same sites that we inventoried in 2018.

It appears that the disease was well established when we first inventoried trees in 2015, and that mortality and canker incidence increased in a coordinated manner between 2015 and 2018 (Fig 9). The CAFI network provides the longest record of mortality for permanent plots and shows a steady increase in mortality since 2000 (Fig 10). Unfortunately, we lack data on earlier endemic contributions to mortality; however, the rapid rise in proportion of dead trees, and increase in variability among sites that parallels the variability of canker incidence, suggest that the canker has played a major role in aspen mortality over the past two decades.

### Mechanisms underlying sudden aspen decline syndrome

In Alaska, sudden aspen decline appears to involve interactions among drought, the fungal canker, and the aspen leaf miner (*Phyllocnistis populiella)* that affect C metabolism and plant water balance to shift aspen beyond its hydraulic safety zone. In addition to the influence of DBH and stand structure on canker incidence (Figs 6 and 7), variability in spring and summer climate among sites contributed to models characterizing the patterning of the disease across the landscape. In particular, the consistent influence of increases in VPD on higher canker incidence point to the net interactive effects of plant and canker responses to temperature, vapor pressure and soil moisture (Figs 8, S1 & S2).

Similar to most other woody angiosperms in temperate to semi-arid environments, aspen operates within a narrow safety margin representing a tradeoff between hydraulic efficiency and risk to drought-induced hydraulic failure [28,57–59]. Hydraulic failure due to xylem embolism during drought is caused by air entering vessels through intervessel pit membranes, often from older vessels [34]. The thickness and porosity of pit membranes are key morphological traits influencing the probability of this air intrusion [60,61]. Despite having comparatively small-diameter vessels, aspen achieves high leaf specific conductance by maintaining lower leaf area to sap wood ratios [33], which contributes to aspen dominating drier habitats than Alaska paper birch, the only other hardwood species found throughout interior Alaskan uplands [62]. Hydraulic efficiency, photosynthesis and growth rate are positively intercorrelated in aspen [63]; however, aspen maintains only 1–2 years of active xylem in rapidly growing branches because of pit membrane degradation [34]. Thus, even a small percentage of embolized 2-yr old xylem driven by drought-insect-pathogen interactions would have large impacts on hydraulic efficiency and associated mortality of aspen. Increased incidence of freeze-thaw events over the past decade in interior Alaska may have also contributed to this mortality syndrome since the loss of hydraulic conductivity due to xylem embolism as cells thaw under tension is known to be correlated with the number of freeze thaw events [33].

Aspen leaf miner (ALM) accelerates leaf water loss by increasing cuticular permeability to evaporation while at the same time reducing photosynthesis via stomatal disruption and potentially chlorophyll degradation [42]. Drought-induced declines in C metabolism, growth, and water balance [29] have likely contributed to the sustained ALM outbreak, via direct and interactive effects on aspen stress and insect survival [40–42]. Aspen produces phenolic glycosides as a direct defense against the ALM, but also produces extrafloral nectaries, an indirect defense that attracts predators such as parasitoid wasps and mites that feed on the miner larvae [64,65]. These collective C costs coupled with the direct effects of the sustained leaf mining on plant water balance likely contribute to mortality through the strong feedbacks between C starvation and hydraulic failure [28,29]. We recorded high ALM damage on all trees inventoried, and thus, were unable to statistically uncouple interactions between the ALM and canker outbreaks. However, because insects and pathogens interact and are known to both influence and respond to changes in plant C and water balance, it is likely that the ALM has contributed to the outbreak of the canker.

Fungal cankers both respond to and exacerbate vulnerability to C starvation and hydraulic failure, and the reliance on only a few years of xylem makes aspen particularly vulnerable to the direct and indirect effects of fungal cankers. Occlusion of pit membranes by fungal hyphae may increase vulnerability to drought-induced cavitation and/or prevent refilling of cavitated vessels during the growing season [66,67]. Experimental fungal canker inoculations in *Populus beijingensis* have been shown to trigger the up-regulation of immune response genes, and the down-regulation of genes related to chemical defense, hydraulic functioning and the metabolism and transport of carbohydrates [36]. Because water stress reduces phloem C transport and carbohydrate supply necessary for osmoregulation and vessel refilling [28], it seems likely that the effects of drought, ALM and canker are triggering feedbacks between plant C and hydraulic stress. Our observations that the highest canker incidence and mortality rates are found on smaller diameter trees in older stands, while younger trees in younger stands appear virtually immune to the disease supports the notion that mortality is driven by C starvation linked with hydraulic failure. Smaller-diameter trees in intermediate-age and older stands have reduced growth rates and defenses due to reductions in light and likely soil water availability [68]. Although younger trees in early-successional stands have ALM, they are likely growing closer to their maximum relative growth rate with a more actively up-regulated defensive system. We suspect that as stands age, all trees become more vulnerable to canker infection because of the effects of increasing LAI on declining soil water content. How belowground C partitioning responds to drought and is influenced by C starvation in aging aspen stands are important but poorly understood factors influencing these dynamics [69–71].

### Ecosystem level consequences of aspen mortality

Warmer early-season temperatures coupled with prolonged late summer drought are leading to larger and more severe wildfires throughout interior Alaska that are favoring a shift from black spruce to forests dominated by Alaska paper birch and aspen [72,73]. Paleoecological studies indicate that the current fire regime is novel in the context of interior Alaskan fires over the past 10,000 years [74] and is likely to persist throughout the 21^st^ century [75,76], although the amount of area burned may stabilize by mid-century due to conversion of the landscape to hardwood forests [77]. Rapid litter decay in deciduous stands and the absence of an insulating moss layer entrain plant-soil-microbial feedbacks that influence long-term trajectories of net primary production, nutrient cycling and C storage [78–80]. These fire-driven shifts to a more deciduous landscape are predicted to have negative feedbacks to climate warming through reduced fire frequency and increased albedo and NPP relative to coniferous forests [81]. Thus, widespread aspen mortality throughout the region has significant consequences for ecosystem function, successional dynamics, and changing disturbance regimes, particularly on sites too dry for Alaska paper birch.

Across interior Alaska, most forests with an aspen-dominated canopy contain at least some slow-growing white spruce and/or black spruce trees in the sub-canopy, and many of the aspen stands we inventoried were a mixture of aspen and conifers. Although the successional models developed decades ago predict that these mixed stands will eventually transition to conifer dominance, their fate has become less certain, and dependent on a number of factors such as moose herbivory on aspen or snowshoe hare herbivory on small conifers [82,83]. In addition, as an intensified fire regime increases fire probability in deciduous stands, perhaps promoted by a high proportion of dead aspen, conifer seedlings and saplings will be killed before they produce viable seed [84]. However, in stands where aspen mortality is high, reaching for example near or over 60% at many of the BNZ LTER Gerstle River stands (Fig 9B), black spruce may experience a growth release triggered by an opening of the aspen canopy. Whether these stands return to black spruce dominance before reburning is dependent on whether they reburn prior to producing seed [84], but current rates of aspen mortality could shift these stands back to the black spruce-moss stand types rather than remaining on the deciduous pathway.

## Supporting information

S1 Fig Climate data for 6 ecoregions sampled. (DOCX)

S2 Fig Relationships between monthly precipitation and vapor pressure deficit for 88 study sites. (DOCX)

S3 Fig Soil moisture (volumetric water content) at 10 cm and 20 cm depths from long-term monitoring at BNZ LTER FP2A, a 105 year old upland forest dominated by a mix of Alaska paper birch, trembling aspen, and white spruce, and BNZ LTER FP1A, a 37 year old upland forest dominated by a mix of Alaska paper birch and trembling aspen. (DOCX)

S1 Table Logistic regression parameter estimates for models incorporating climate variables. (DOCX)

S1 Document Diagnostic features of canker lesion characteristic of “Aspen running canker” and testing Koch’s postulates. (DOCX)

## References

[1] Seidl R, Klonner G, Rammer W, Essl F, Moreno A, Neumann M, et al. Invasive alien pests threaten the carbon stored in Europe’s forests. Nature Communications. 2018;9:1626(1). 10.1038/s41467-018-04096-w 29691396 PMC5915461

[2] Wyka SA, Munck IA, Brazee NJ, Broders KD. Response of eastern white pine and associated foliar, blister rust, canker and root rot pathogens to climate change. Forest Ecology and Management. 2018;423:18–26. 10.1016/j.foreco.2018.03.011

[3] Kautz M, Anthoni P, Meddens AJH, Pugh TAM, Arneth A. Simulating the recent impacts of multiple biotic disturbances on forest carbon cycling across the United States. Global Change Biology. 2018;24(5):2079–92. 10.1111/gcb.13974 29105233

[4] Ghelardini L, Luchi N, Pecori F, Pepori AL, Danti R, Della Rocca G, et al. Ecology of invasive forest pathogens. Biological Invasions. 2017;19(11):3183–200. 10.1007/s10530-017-1487-0

[5] Hennon P, Frankel S, Woods A, Worrall J, Norlander D, Zambino P, et al. A framework to evaluate climate effects on forest tree diseases. Forest Pathology. 2020;e12649:1–10. 10.111/efp.12649

[6] Wang J, Hu M, Wang J, Qi J, Han Z, Wang G, et al. Reconstitution and structure of a plant NLR resistosome conferring immunity. Science. 2019;364:eaav5870(6435). 10.1126/science.aav5870 30948527

[7] Dangl JL, Jones JDG. Plant pathogens and integrated defence responses to infection. Nature. 2001;411(6839):826–33. 10.1038/35081161 11459065

[8] Jones JDG, Vance RE, Dangl JL. Intracellular innate immune surveillance devices in plants and animals. Science. 2016;354(6316). 10.1126/science.aaf6395 27934708

[9] Mukhtar MS, Carvunis AR, Dreze M, Epple P, Steinbrenner J, Moore J, et al. Independently evolved virulence effectors converge onto hubs in a plant immune system network. Science. 2011;333(6042):596–601. 10.1126/science.1203659 21798943 PMC3170753

[10] Ostry ME, Anderson NA. Genetics and ecology of the Entoleuca mammata-Populus pathosystem: Implications for aspen improvement and management. Forest Ecology and Management. 2009;257(2):390–400. 10.1016/j.foreco.2008.09.053

[11] Ostry ME, Anderson NA. Infection of *Populus tremuloides* by *Hypoxylon mammatum* ascospores through *Saperda inornata* galls. Canadian Journal of Forest Research. 1995;25(5):813–6. 10.1139/x95-088

[12] Panzavolta T, Panichi A, Bracalini M, Croci F, Benigno A, Ragazzi A, et al. Tree pathogens and their insect-mediated transport: Implications for oak tree die-off in a natural park area. Global Ecology and Conservation. 2018;15:e00437. 10.1016/j.gecco.2018.e00437

[13] Kaczynski KM, Cooper DJ, Jacobi WR. Interactions of sapsuckers and *Cytospora* canker can facilitate decline of riparian willows. Botany. 2014;92(7):485–93. 10.1139/cjb-2014-0019

[14] Worrall JJ, Egeland L, Eager T, Mask RA, Johnson EW, Kemp PA, et al. Rapid mortality of *Populus tremuloides* in southwestern Colorado, USA. Forest Ecology and Management. 2008;255:686–96.

[15] Worrall JJ, Keck AG, Marchetti SB. *Populus tremuloides* stands continue to deteriorate after drought-incited sudden aspen decline. Canadian Journal of Forest Research. 2015;45(12):1768–74. 10.1139/cjfr-2015-0225 WOS:000365335600011.

[16] Dudley MM, Burns KS, Jacobi WR. Aspen mortality in the Colorado and southern Wyoming Rocky Mountains: Extent, severity, and causal factors. Forest Ecology and Management. 2015;353:240–59. 10.1016/j.foreco.2015.06.002

[17] Agne MC, Beedlow PA, Shaw DC, Woodruff DR, Lee EH, Cline SP, et al. Interactions of predominant insects and diseases with climate change in Douglas-fir forests of western Oregon and Washington, U.S.A. Forest Ecology and Management. 2018;409:317–32. 10.1016/j.foreco.2017.11.004 29290644 PMC5746199

[18] Rogers BM, Solvik K, Hogg EH, Ju J, Masek JG, Michaelian M, et al. Detecting early warning signals of tree mortality in boreal North America using multiscale satellite data. Global Change Biology. 2018;24(6):2284–304. 10.1111/gcb.14107 29481709

[19] Vaughn NR, Asner GP, Brodrick PG, Martin RE, Heckler JW, Knapp DE, et al. An approach for high-resolution mapping of Hawaiian Metrosideros Forest mortality using Laser-Guided Imaging Spectroscopy. Remote Sensing. 2018;10:502(4). 10.3390/rs10040502

[20] He Y, Chen G, Potter C, Meentemeyer RK. Integrating multi-sensor remote sensing and species distribution modeling to map the spread of emerging forest disease and tree mortality. Remote Sensing of Environment. 2019;231:111238. 10.1016/j.rse.2019.111238

[21] Imanyfar S, Hasanlou M, Mirzaei Zadeh V. Mapping oak decline through long-term analysis of time series of satellite images in the forests of Malekshahi, Iran. International Journal of Remote Sensing. 2019;40(23):8705–26. 10.1080/01431161.2019.1620375

[22] Hogg EH, Brandt JP, Michaelian M. Impacts of a regional drought on the productivity, dieback, and biomass of western Canadian aspen forests. Canadian Journal of Forest Research. 2008;38(6):1373–84. 10.1139/X08-001

[23] Michaelian M, Hogg EH, Hall RJ, Arsenault E. Massive mortality of aspen following severe drought along the southern edge of the Canadian boreal forest. Global Change Biology. 2011;17(6):2084–94. 10.1111/j.1365-2486.2010.02357.x

[24] Worrall JJ, Rehfeldt GE, Hamann A, Hogg EH, Marchetti SB, Michaelian M, et al. Recent declines of *Populus tremuloides* in North America linked to climate. Forest Ecology and Management. 2013;299:35–51. 10.1016/j.foreco.2012.12.033

[25] Chen L, Huang JG, Alam SA, Zhai L, Dawson A, Stadt KJ, et al. Drought causes reduced growth of trembling aspen in western Canada. Global Change Biology. 2017;23(7):2887–902. 10.1111/gcb.13595 28121057

[26] Singer JA, Turnbull R, Foster M, Bettigole C, Frey BR, Downey MC, et al. Sudden aspen decline: A review of pattern and process in a changing climate. Forests. 2019;10:671. 10.3390/f10080671

[27] Lindroth RL, St. Clair SB. Adaptations of quaking aspen (*Populus tremuloides* Michx.) for defense against herbivores. Forest Ecology and Management. 2013;299:14–21.

[28] Sevanto S, McDowell NG, Dickman LT, Pangle R, Pockman WT. How do trees die? A test of the hydraulic failure and carbon starvation hypotheses. Plant, Cell and Environment. 2014;37(1):153–61. 10.1111/pce.12141 23730972 PMC4280888

[29] McDowell NG, Beerling DJ, Breshears DD, Fisher RA, Raffa KF, Stitt M. The interdependence of mechanisms underlying climate-driven vegetation mortality. Trends in Ecology and Evolution. 2011;26(10):523–32. 10.1016/j.tree.2011.06.003 21802765

[30] Anderegg WRL, Anderegg LDL, Berry JA, Field CB. Loss of whole-tree hydraulic conductance during severe drought and multi-year forest die-off. Oecologia. 2014;175(1):11–23. 10.1007/s00442-013-2875-5 24394863

[31] Anderegg WRL, Flint A, Huang CY, Flint L, Berry JA, Davis FW, et al. Tree mortality predicted from drought-induced vascular damage. Nature Geoscience. 2015;8(5):367–71. 10.1038/ngeo2400

[32] Anderegg WRL, Plavcová L, Anderegg LDL, Hacke UG, Berry JA, Field CB. Drought’s legacy: Multiyear hydraulic deterioration underlies widespread aspen forest die-off and portends increased future risk. Global Change Biology. 2013;19(4):1188–96. 10.1111/gcb.12100 23504895

[33] Sperry JS, Nichols KL, Sullivan JEM, Eastlack SE. Xylem embolism in ring-porous, diffuse-porous, and coniferous trees of northern Utah and interior Alaska. Ecology. 1994;75(6):1736–52. ISI:A1994PE26700021.

[34] Sperry JS, Perry AH, Sullivan JEM. Pit membrane degradation and air-embolism formation in ageing xylem vessels of *Populus tremuloides* michx. Journal of Experimental Botany. 1991;42(11):1399–406. 10.1093/jxb/42.11.1399

[35] Tai X, Mackay DS, Anderegg WRL, Sperry JS, Brooks PD. Plant hydraulics improves and topography mediates prediction of aspen mortality in southwestern USA. New Phytologist. 2017;213(1):113–27. 10.1111/nph.14098 27432086

[36] Li P, Liu W, Zhang Y, Xing J, Li J, Feng J, et al. Fungal canker pathogens trigger carbon starvation by inhibiting carbon metabolism in poplar stems. Scientific Reports. 2019;9(1). 10.1038/s41598-019-46635-5 31300723 PMC6626041

[37] Bieniek PA, Walsh JE, Thoman RL, Bhatt US. Using climate divisions to analyze variations and trends in Alaska temperature and precipitation. Journal of Climate. 2014;27(8):2800–18. 10.1175/jcli-d-13-00342.1 WOS:000334017400002.

[38] Thoman R, Walsh J. Alaska’s changing environment: documenting Alaska’s physical and biological changes through observations 2019.

[39] Cahoon SMP, Sullivan PF, Brownlee AH, Pattison RR, Andersen HE, Legner K, et al. Contrasting drivers and trends of coniferous and deciduous tree growth in interior Alaska. Ecology. 2018;99(6):1284–95. 10.1002/ecy.2223 29569245

[40] Wagner D, DeFoliart L, Doak P, Schneiderheinze J. Impact of epidermal leaf mining by the aspen leaf miner (*Phyllocnistis populiella*) on the growth, physiology, and leaf longevity of quaking aspen. Oecologia. 2008;157(2):259–67. 10.1007/s00442-008-1067-1 WOS:000257956000008. 18523809

[41] Wagner D, Doak P. Long-term impact of a leaf miner outbreak on the performance of quaking aspen. Canadian Journal of Forest Research-Revue Canadienne De Recherche Forestiere. 2013;43(6):563–9. 10.1139/cjfr-2012-0486 WOS:000320231100006.

[42] Wagner D, Wheeler J, Burr S. The leaf miner *Phyllocnistis populiella* negatively impacts water relations in aspen. Tree Physiology. 2020;40:580–90. 10.1093/treephys/tpz109 31728531

[43] Boyd MA, Berner LT, Doak P, Goetz SJ, Rogers BM, Wagner D, et al. Impacts of climate and insect herbivory on productivity and physiology of trembling aspen (*Populus tremuloides*) in Alaskan boreal forests. Environmental Research Letters. 2019;14(8). 10.1088/1748-9326/ab215f

[44] USDA. Forest health conditions in Alaska—2019. In: USDA, editor.: USDA, Washington, DC; 2020.

[45] Trugman AT, Medvigy D, Anderegg WRL, Pacala SW. Differential declines in Alaskan boreal forest vitality related to climate and competition. Global Change Biology. 2018;24(3):1097–107. 10.1111/gcb.13952 29055122

[46] Crous P, Groenewald J, Adams G, Winton L. Fungal planet no. 1141. Persoonia—Molecular Phylogeny and Evolution of Fungi. 2020;45.

[47] Winton L, Adams G, Ruess R. Determining the causal agent of the aspen running canker disease in Alaska. Canadian Journal of Forest Pathology. (*in review*).

[48] Malone T, Liang J, Packee E. Cooperative Alaska Forest Inventory. Department of Agriculture, Pacific Northwest Research Station, 2009 PNW-GTR-785 Contract No.: PNW-GTR-785.

[49] R Development Core Team. R: A Language and Environment for Statistical Computing. Vienna, Austria: R Foundation for Statistical Computing; 2017.

[50] SAS. SAS University Edition. 4 ed. Cary, NC: SAS Institute, Inc.; 2018.

[51] Daly C, Halbleib M, Smith JI, Gibson WP, Doggett MK, Taylor GH, et al. Physiographically sensitive mapping of climatological temperature and precipitation across the conterminous United States. International Journal of Climatology. 2008;28(15):2031–64. 10.1002/joc.1688

[52] Nicklen EF, Roland CA, Csank AZ, Wilmking M, Ruess RW, Muldoon LA. Stand basal area and solar radiation amplify white spruce climate sensitivity in interior Alaska: Evidence from carbon isotopes and tree rings. Global Change Biology. 2019;25(3):911–26. 10.1111/gcb.14511 30408264

[53] Sinclair WA, Lyon HH. Diseases of trees and shrubs. 2nd ed. Ithaca, New York, USA: Cornell University Press; 2005. 660 p.

[54] Lian X, Piao S, Li LZX, Li Y, Huntingford C, Ciais P, et al. Summer soil drying exacerbated by earlier spring greening of northern vegetation. Science Advances. 2020;6(1):eaax0255. 10.1126/sciadv.aax0255 31922002 PMC6941915

[55] Buermann W, Bikash PR, Jung M, Burn DH, Reichstein M. Earlier springs decrease peak summer productivity in North American boreal forests. Environmental Research Letters. 2013;8(2). 10.1088/1748-9326/8/2/024027 WOS:000321425100031.

[56] Marchetti SB, Worrall JJ, Eager T. Secondary insects and diseases contribute to sudden aspen decline in southwestern Colorado, USA. Canadian Journal of Forest Research. 2011;41(12):2315–25. 10.1139/X11-106

[57] Choat B, Jansen S, Brodribb TJ, Cochard H, Delzon S, Bhaskar R, et al. Global convergence in the vulnerability of forests to drought. Nature. 2012;491(7426):752–5. 10.1038/nature11688 23172141

[58] Gleason SM, Westoby M, Jansen S, Choat B, Hacke UG, Pratt RB, et al. Weak tradeoff between xylem safety and xylem-specific hydraulic efficiency across the world’s woody plant species. New Phytologist. 2016;209(1):123–36. 10.1111/nph.13646 26378984

[59] Maherali H, Pockman WT, Jackson RB. Adaptive variation in the vulnerability of woody plants to xylem cavitation. Ecology. 2004;85(8):2184–99. 10.1890/02-0538

[60] Lens F, Tixier A, Cochard H, Sperry JS, Jansen S, Herbette S. Embolism resistance as a key mechanism to understand adaptive plant strategies. Current Opinion in Plant Biology. 2013;16(3):287–92. 10.1016/j.pbi.2013.02.005 23453076

[61] Jansen S, Choat B, Pletsers A. Morphological variation of intervessel pit membranes and implications to xylem function in angiosperms. American Journal of Botany. 2009;96(2):409–19. 10.3732/ajb.0800248 21628196

[62] Viereck LA, Dyrness CT, Van Cleve K, Foote MJ. Vegetation, soils, and forest productivity in selected forest types in interior Alaska. Canadian Journal of Forest Research. 1983;13:703–20.

[63] Hajek P, Leuschner C, Hertel D, Delzon S, Schuldt B. Trade-offs between xylem hydraulic properties, wood anatomy and yield in *Populus*. Tree Physiology. 2014;34(7):744–56. 10.1093/treephys/tpu048 25009155

[64] Young B, Wagner D, Doak P, Clausen T. Induction of phenolic glycosides by quaking aspen (*Populus tremuloides*) leaves in relation to extrafloral nectaries and epidermal leaf mining. Journal of Chemical Ecology. 2010;36(4):369–77. 10.1007/s10886-010-9763-9 WOS:000276430700003. 20354896

[65] Young B, Wagner D, Doak P, Clausen T. Within-plant distribution of phenolic glycosides and extrafloral nectaries in trembing aspen (*Populus tremuloides*; Salicaceae). American Journal of Botany. 2010;97(4):601–10. 10.3732/ajb.0900281 WOS:000276045500007. 21622422

[66] Madar Z, Solel Z, Sztejnberg A. The effect of *Diplodia pinea* f.sp. *cupressi* and *Seiridium cardinale* on water flow in cypress branches. Physiological and Molecular Plant Pathology. 1990;37(5):389–98. 10.1016/0885-5765(90)90121-D

[67] Love DM, Sperry JS. In situ embolism induction reveals vessel refilling in a natural aspen stand. Tree Physiology. 2018;38(7):1006–15. 10.1093/treephys/tpy007 29509942

[68] Najar A, Landhäusser SM, Whitehill JGA, Bonello P, Erbilgin N. Reserves accumulated in non-photosynthetic organs during the previous growing season drive plant defenses and growth in aspen in the subsequent growing season. Journal of Chemical Ecology. 2014;40(1):21–30. 10.1007/s10886-013-0374-0 24363094

[69] Landhäusser SM, Lieffers VJ. Defoliation increases risk of carbon starvation in root systems of mature aspen. Trees—Structure and Function. 2012;26(2):653–61. 10.1007/s00468-011-0633-z

[70] Giardina CP, Litton CM, Crow SE, Asner GP. Warming-related increases in soil CO2 effux are explained by increased below-ground carbon flux. Nature Climate Change. 2014;4(9):822–7. 10.1038/nclimate2322 WOS:000341569700027.

[71] Litton CM, Giardina CP. Below-ground carbon flux and partitioning: global patterns and response to temperature. Functional Ecology. 2008;22:941–54.

[72] Johnstone JF, Hollingsworth TN, Chapin FS, III, Mack MC. Changes in fire regime break the legacy lock on successional trajectories in the Alaskan boreal forest. Global Change Biology. 2010;16:1281–95.

[73] Barrett K, McGuire AD, Hoy EE, Kasischke ES. Potential shifts in dominant forest cover in interior Alaska driven by variations in fire severity. Ecological Applications. 2011;21(7):2380–96. WOS:000296139200004. 10.1890/10-0896.1 22073630

[74] Kelly R, Chipman ML, Higuera PE, Stefanova I, Brubaker LB, Hu FS. Recent burning of boreal forests exceeds fire regime limits of the past 10,000 years. Proceedings of the National Academy of Sciences of the United States of America. 2013;110(32):13055–60. 10.1073/pnas.1305069110 WOS:000322771100054. 23878258 PMC3740857

[75] Balshi MS, McGuire AD, Duffy P, Kicklighter DW, Melillo JM. Vulnerability of carbon storage in North American boreal forests to wildfires during the 21st Century. Global Change Biology. 2009;15:1491–510.

[76] Mann DH, Rupp TS, Olson MA, Duffy PA. Is Alaska’s boreal forest now crossing a major ecological threshold? Arctic Antarctic and Alpine Research. 2012;44(3):319–31. 10.1657/1938-4246-44.3.319 WOS:000307990800006.

[77] Wylie B, Pastick NJ, Johnson K, Bliss N, Genet H, editors. Soil carbon and permafrost estimates and susceptibility in Alaska. Chapter3. Washington, D.C. (In press): U.S. Geological Survey Professional Paper; 2016.

[78] Johnstone JF, Chapin FS, Hollingsworth TN, Mack MC, Romanovsky VE, Turetsky MR. Fire, climate change, and forest resilience in interior Alaska. Canadian Journal of Forest Research. 2010;40:1302–12.

[79] Melvin AM, Mack MC, Johnstone JF, McGuire AD, Genet H, Schuur EAG. Differences in ecosystem carbon distribution and nutrient cycling linked to forest tree species composition in a mid-successional boreal forest. Ecosystems. 2015;18(8):1472–88. 10.1007/s10021-015-9912-7 WOS:000365096400013.

[80] Alexander HD, Mack MC. A canopy shift in interior Alaskan boreal forests: consequences for above- and belowground carbon and nitrogen pools during post-fire succession. Ecosystems. 2016;19:98–114.

[81] Pastick NJ, Duffy P, Genet H, Rupp TS, Wylie BK, Johnson KD, et al. Historical and projected trends in landscape drivers affecting carbon dynamics in Alaska. Ecological Applications. 2017;27(5):1383–402. 10.1002/eap.1538 28390104

[82] Olnes J, Kielland K, Genet H, Ruess RW. Post-fire deciduous canopies drive patterns in snowshoe hare herbivory of regenerating black spruce. Canadian Journal of Forest Research. 2019;49:1392–2399.

[83] Conway AJ, Johnstone JF. Moose alter the rate but not the trajectory of forest canopy succession after low and high severity fire in Alaska. Forest Ecology and Management. 2017;391:154–63.

[84] Johnstone JF, Allen CD, Franklin JF, Frelich LE, Harvey BJ, Higuera PE, et al. Changing disturbance regimes, ecological memory, and forest resilience. Frontiers in Ecology and the Environment. 2016;14(7):369–78. 10.1002/fee.1311 WOS:000382527900016.

