# Correction: Widespread mortality of trembling aspen (*Populus tremuloides*) throughout interior Alaskan boreal forests resulting from a novel canker disease

**DOI:** 10.1371/journal.pone.0253996

**Published:** 2021-06-25

**Authors:** Roger W. Ruess, Loretta M. Winton, Gerard C. Adams

In Fig 2, the label “r2 = 0.36, P < 0.0001” refers to Panel A, and the label “r2 = 0.28, P < 0.0001” refers to panel B. Please see the correct Fig 2 here.

**Fig 2:**
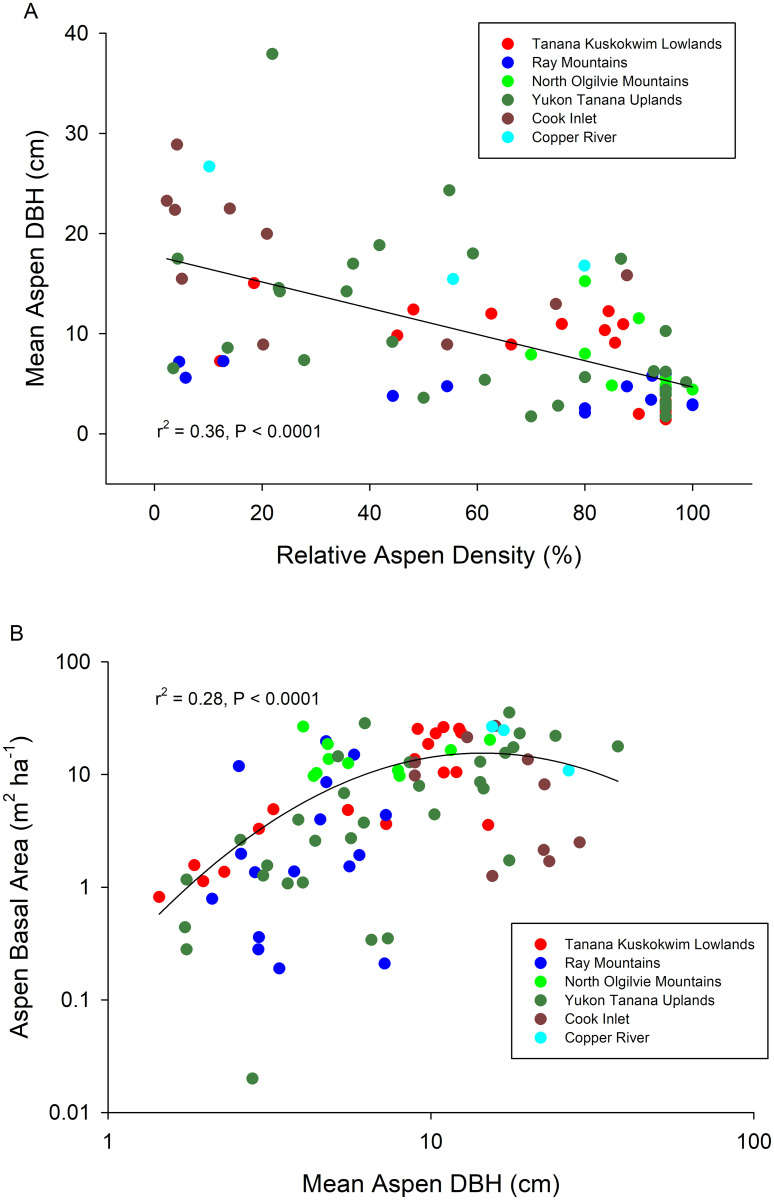
Relationships between stand structural characteristics across the 88 study sites. Mean aspen DBH vs. percentage of trees that were aspen (= relative aspen density) across stands where aspen grew with white spruce, black spruce, Alaskan paper birch, or a combination (A) and aspen basal area vs. mean aspen DBH (B) across study sites.

## References

[1] Ruess RW, Winton LM, Adams GC (2021) Widespread mortality of trembling aspen (*Populus tremuloides*) throughout interior Alaskan boreal forests resulting from a novel canker disease. PLoS ONE 16(4): e0250078. doi: 10.1371/journal.pone.0250078 33831122 PMC8032200

