# Supplementary material for: Widespread mortality of trembling aspen (Populus tremuloides) throughout interior Alaskan boreal forests resulting from a novel canker disease
Source: PLoS One. 2021 Apr 8;16(4):e0250078. doi: 10.1371/journal.pone.0250078 (PMC8032200; doi:10.1371/journal.pone.0250078)
Supplement: S2 Fig — (DOCX) [file pone.0250078.s002.docx]

**S4 Figure. Relationships between monthly precipitation and vapor pressure deficit for 88 study sites.** Data obtained from SNAP (https://www.snap.uaf.edu/tools/data-downloads).
