# Supplementary material for: Widespread mortality of trembling aspen (Populus tremuloides) throughout interior Alaskan boreal forests resulting from a novel canker disease
Source: PLoS One. 2021 Apr 8;16(4):e0250078. doi: 10.1371/journal.pone.0250078 (PMC8032200; doi:10.1371/journal.pone.0250078)
Supplement: S3 Fig — (DOCX) [file pone.0250078.s003.docx]

**S5 Figure. Soil moisture (volumetric water content) at 10 cm and 20 cm depths from long-term monitoring at BNZ LTER FP2A, a 105 year old upland forest dominated by a mix of Alaska paper birch, trembling aspen, and white spruce, and BNZ LTER FP1A, a 37 year old upland forest dominated by a mix of Alaska paper birch and trembling aspen.** Hourly data were first averaged to produce monthly means. Data presented here are monthly means ± 1 SE averaged over a 17-year period (2003-2019). More information about these stands (located in the Tanana Kuskokwim Lowlands ecoregion) and other BNZ LTER research sites can be found at http://www.lter.uaf.edu/data/data-catalog.
