# Supplementary material for: Widespread mortality of trembling aspen (Populus tremuloides) throughout interior Alaskan boreal forests resulting from a novel canker disease
Source: PLoS One. 2021 Apr 8;16(4):e0250078. doi: 10.1371/journal.pone.0250078 (PMC8032200; doi:10.1371/journal.pone.0250078)
Supplement: S1 Table — (DOCX) [file pone.0250078.s004.docx]

**S3 Table. Logistic regression parameter estimates for models incorporating climate variables**. Parameter estimates (α and β_i_) for best mixed-effects logistic regression models fitted separately for each month evaluating the effects of stand structure and monthly climate on canker incidence with site within ecoregion as the random effect: Logit (π) = log (π/(1- π)) = α + β_1_ **DBH* + β_2_ **Relative Aspen Density* + β_3_ **Aspen Basal Area* + β_4_ **Mean Aspen DBH* + β_5_ **VPDmonth*_._. DBH = tree diameter (cm), RAD = relative aspen density (%), ABA = aspen basal area (m^2^ ha^-1^), MADBH = mean aspen DBH (cm), VPDmonth = monthly VPD (hPa). Estimate standard errors are listed in parentheses; marginal (*R^2^_m_*) and conditional (*R^2^_c_*) values for models describe variance explained by fixed and fixed+random effects, respectively.

| Month | intercept | DBH | RAD | ABA | MADBH | VPD | *R^2^_m_* | *R^2^_c_* |
| --- | --- | --- | --- | --- | --- | --- | --- | --- |
| MAY | -4.352 (1.235) | -0.177 (0.008) | -0.021 (0.007) | 0.086 (0.022) | 0.080 (0.037) | 0.653 (0.233) | 0.20 | 0.47 |
| JUN | -3.846 (1.277) | -0.177 (0.008) | -0.021 (0.007) | 0.087 (0.022) | 0.087 (0.038) | 0.309 (0.153) | 0.17 | 0.47 |
| JUL | -3.882 (1.327) | -0.177 (0.008) | -0.021 (0.007) | 0.083 (0.022) | 0.088 (0.038) | 0.385 (0.215) | 0.17 | 0.48 |
| AUG | -4.251 (1.638) | -0.177 (0.008) | -0.022 (0.007) | 0.074 (0.023) | 0.081 (0.038) | 0.837 (0.324) | 0.17 | 0.50 |
