# Supplementary material for: Widespread mortality of trembling aspen (Populus tremuloides) throughout interior Alaskan boreal forests resulting from a novel canker disease
Source: PLoS One. 2021 Apr 8;16(4):e0250078. doi: 10.1371/journal.pone.0250078 (PMC8032200; doi:10.1371/journal.pone.0250078)
Supplement: S1 Document — (DOCX) [file pone.0250078.s005.docx]

**S1 Document. Diagnostic features of canker lesion characteristic of “Aspen running canker”**
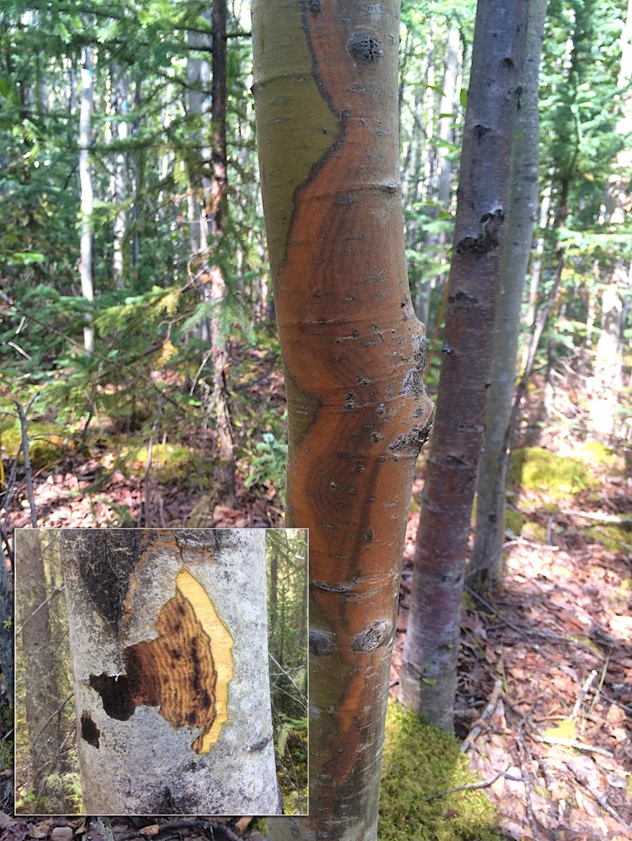
 **and testing Koch’s postulates.** Working with Dr. Pedro W. Crous, Professor of Fungal Biodiversity, Utrecht University, we have confirmed the pathogen is novel to science and have named the yeast-like fungus (*Neodothiora* *populina* Crous, G.C. Adams, & Winton) [46]. Fruiting of the pathogen has not been seen on the canker margins of dying and dead aspen, the cankers are diffuse with distinct margins and are often extensive in size, and length commonly reaches over 1 m. Additional characterizations are described in the text. Picture on the right shows symptoms of a naturally infected aspen running canker lesion on trembling aspen in Interior Alaska. Inset: Debarked lesion showing successive advances of brown fungal-killed tissue. Photo by Lori Winton [47]. Canker breadth varies and eventually girdles smaller diameter trees. On mature, large diameter trees (i.e., ~80-year-old trees) with thick bark, canker breadth can be difficult to determine. Cankers seldom extend from the soil and do not appear to be associated with insect boring, branch breakage, moose browsing or bark stripping, or other noticeable injury to bark or epidermis. Once infected, the canker can spread rapidly within a tree; we noticed trees with dead and dying current year’s leaves and well-developed canker, and in several instances, canker breadth expanded by > 3 cm within a month.

Our study confirming Koch’s postulates for this pathogen is described elsewhere [47]. Briefly, isolations of putative causal agents were collected using sterilized borer taken through the inner bark, cambium, and outer sapwood along canker margins of trees, aseptically placed on selective media, and incubated. After incubation, cultures were grouped by visible cultural characteristics and DNA was isolated and purified from several representative cultures for each group. Species determinations were based on DNA barcoding and microscopic examinations. Taxonomically informative partial gene sequences of relevant fungal species identified were deposited as accessions in the NCBI GenBank database. Inoculations for pathogenicity assessment were conducted on replicate cut logs that were cultured in environmental chambers, and on live trees growing in replicated field locations. Quantitative measures of developing lesions on growth chamber logs after 1 month and on field trees after 8 and 12 months showed strong differences in pathogenicity of isolates. Reisolations from inoculated logs and reidentification of the cultures confirmed Koch’s postulates by identifying *Neodothiora* *populina* as the primary disease causal agent.
